# Biofunctional Pectin Derived from Pomelo Peel: Structural Insights and Neuro–Gut Protective Mechanisms in Zebrafish under Bisphenol AF-Induced Neurotoxicity

**DOI:** 10.34133/research.1263

**Published:** 2026-05-07

**Authors:** Bowen Yan, Junping Deng, Xinyu Hu, Shuo Tang, Qiang Yong, Jie Gu, Caoxing Huang

**Affiliations:** ^1^ State Key Laboratory for Development and Utilization of Forest Food Resources, Key Lab. of Biomass Energy and Material, International Innovation Center for Forest Chemicals and Materials and Jiangsu Co-Innovation Center of Efficient Processing and Utilization of Forest Resources, Institute of Chemical Industry of Forest Products, Chinese Academy of Forestry, Nanjing 210042, China.; ^2^ State Key Laboratory for the Development and Utilization of Forest Food Resources, Nanjing Forestry University, Nanjing 210037, China.; ^3^ Nanjing Institute of Comprehensive Utilization of Wild Plants, Nanjing 211111, China.; ^4^ Nanjing Institute of Environmental Sciences, Ministry of Ecology and Environment, Nanjing 210042, China.

## Abstract

Pomelo (*Citrus maxima* cv. Wentan) peel represents a major agricultural byproduct rich in pectin with potential applications in functional foods and biomedical materials. This study aimed to elucidate the structure–activity relationship of pomelo peel pectin extracted at different temperatures (100 to 140 °C) and to evaluate its neuro–gut protective effects using a zebrafish model. Pectins obtained under varying thermal conditions were structurally characterized, and their bioactivities were systematically compared. Among them, pectin extracted at 130 °C (P-130) exhibited higher galacturonic acid content, molecular weight, and hydroxyl group abundance. Functionally, P-130 effectively mitigated bisphenol AF-induced toxicity in zebrafish. It significantly improved developmental outcomes, reduced oxidative stress, and preserved neuronal integrity by promoting axonal growth and up-regulating neurodevelopment-related genes. Notably, P-130 regulated the gut–brain axis by restoring neurotransmitter balance (5-hydroxytryptamine and dopamine), attenuating intestinal inflammation, and modulating gut microbiota composition, particularly by enriching beneficial taxa such as Bacteroidetes. These effects were associated with reduced anxiety-like behavior. Histopathological analyses further confirmed its protective roles in cerebellar and intestinal tissues. This study highlights the critical influence of extraction temperature on pectin structure and identifies P-130 as a promising natural agent against environmental toxicants. The findings provide novel insights into the synergistic mechanisms linking antioxidant activity, neuroprotection, and microbiota regulation, offering a scientific basis for the valorization of pomelo peel in developing plant-derived protective strategies.

## Introduction

China produces over half of the world’s pomelo, with an annual output exceeding 5 million tons, and this massive industry simultaneously generates over 2 million tons of peel waste annually [1,2]. Conventional treatment methods, such as landfilling and incineration, waste valuable biomass resources and impose substantial environmental burdens due to the peel’s high organic content [3]. Therefore, developing strategies for the high-value utilization of these agroforestry residues is essential for advancing sustainable agriculture and promoting a circular economy [4]. Increasingly, pomelo peel is no longer regarded as mere waste but recognized as a natural reservoir of bioactive compounds, including pectin, naringin, hesperidin, and various phenolic acids.

Among the bioactive compounds in pomelo peel, pectin is particularly abundant (20% to 30% of dry weight) and suitable for industrial-scale extraction [5]. Pectin exhibits diverse biological activities, including intestinal flora modulation, lipid-lowering effects, glycemic regulation, and heavy-metal chelation [6]. In parallel, flavonoids such as naringin and hesperidin display strong antioxidant, anti-inflammatory, and neuroprotective activities [7], whereas phenolic acids and essential oils provide additional synergistic effects. Despite these promising properties, existing research has primarily focused on the physicochemical characteristics of pomelo peel pectin and its gastrointestinal health benefits [8]. For instance, Wang et al. [9] enzymatically modified pomelo peel pectin and obtained a product that enhanced gut short-chain fatty acid production, increased *Bacteroides* abundance, and inhibited *Escherichia*–*Shigella* growth during in vitro fermentation. Additionally, Zhao et al. [10] isolated a pectic polysaccharide (EPS-2B) from *Euphorbia humifusa*, which dose-dependently reduced lipid accumulation and oxidative stress in L02 cells, indicating its potential as a lipid-lowering agent. In contrast, the potential neuroprotective effects of pectin, particularly against environmental neurotoxins, have not been extensively investigated.

Environmental neurotoxins, including heavy metals, persistent organic pollutants, and air pollutants, impair neural function predominantly through oxidative stress and neuroinflammation [11]. In contrast, plant-derived extracts exert neuroprotection through antioxidant, anti-inflammatory, and metal chelating mechanisms that counteract these toxic pathways [12]. Among persistent organic pollutants, bisphenol A and its structural analog bisphenol AF (BPAF) are of particular concern. BPAF, widely used as a bisphenol A substitute, exhibits greater environmental persistence and bioaccumulation potential due to its trifluoromethyl groups [13]. BPAF readily penetrates the blood–brain barrier and induces neurotoxicity through multiple mechanisms, including oxidative stress, neuroinflammation, neurotransmitter disruption, and neuronal apoptosis [14]. These pathological alterations manifest as neuronal structural damage, aberrant glial activation, and behavioral impairments in zebrafish, thereby establishing BPAF as a reliable agent for modeling neurotoxicity [15]. Pectin has garnered significant interest in its antioxidant, anti-inflammatory, and prebiotic properties, exerting its biological effects through the microbiota–gut–brain (MGB) axis [16]. These characteristics directly counteract BPAF-induced neurotoxic pathways, suggesting that pectin may mitigate BPAF-induced neurotoxicity via MGB-axis-mediated mechanisms. Thus, the BPAF-induced zebrafish model of neural injury provides a robust platform for evaluating the neuroprotective efficacy of natural products, such as pectin.

Zebrafish have emerged as a valuable vertebrate model for natural product research due to their approximately 70% genomic homology with humans and their conserved biological processes, including neural development and signaling pathways [17]. Their transparent embryos enable real-time visualization of neuronal structures, while rapid development, low maintenance costs, and suitability for high-throughput screening markedly enhance research efficiency [17]. Furthermore, diverse behavioral assays (locomotor activity, memory function, and stress responses) and advanced genetic manipulation approaches support both functional and mechanistic investigations [18]. Imbalances in neurotransmitter levels are closely associated with the occurrence and development of anxiety-like behaviors, which can be reliably quantified using zebrafish behavioral. Particularly advantageous is the suitability of zebrafish for MGB axis research, as the bidirectional regulatory mechanisms between the gut and central nervous system are highly conserved, enabling real-time tracking of dietary components and their effects on neural function through in vivo imaging [19]. These features collectively position zebrafish as an optimal model for elucidating the neuroprotective mechanisms of pomelo peel pectin.

Therefore, the present study systematically investigated the protective effects and underlying mechanisms of pomelo peel pectin against BPAF-induced neurotoxicity in zebrafish based on the MGB axis. Specifically, pectin was extracted by hot-water extraction and characterized using multiple analytical techniques, followed by the establishment of a BPAF-induced neurotoxicity model to evaluate its effects on zebrafish neurobehavioral outcomes, including locomotor activity, anxiety-like responses, and cognitive performance. Additionally, neurotransmitter levels (dopamine [DA], serotonin, and acetylcholine) were quantified to determine regulatory effects on synaptic signaling, whereas 16S ribosomal DNA (rDNA) sequencing was performed to investigate possible gut–brain axis interactions. Finally, oxidative stress markers, inflammatory cytokines, and neurotransmitter-related genes were assessed to elucidate the molecular basis of the neuroprotective actions of pectin. Overall, this study not only establishes a sustainable and high-value utilization pathway for pomelo peel in the context of neuroprotection but also broadens the functional scope of pectin.

## Results and Discussion

### Analysis of the glycosyl composition and molecular weight of obtained pectins

**Fig. 1.**
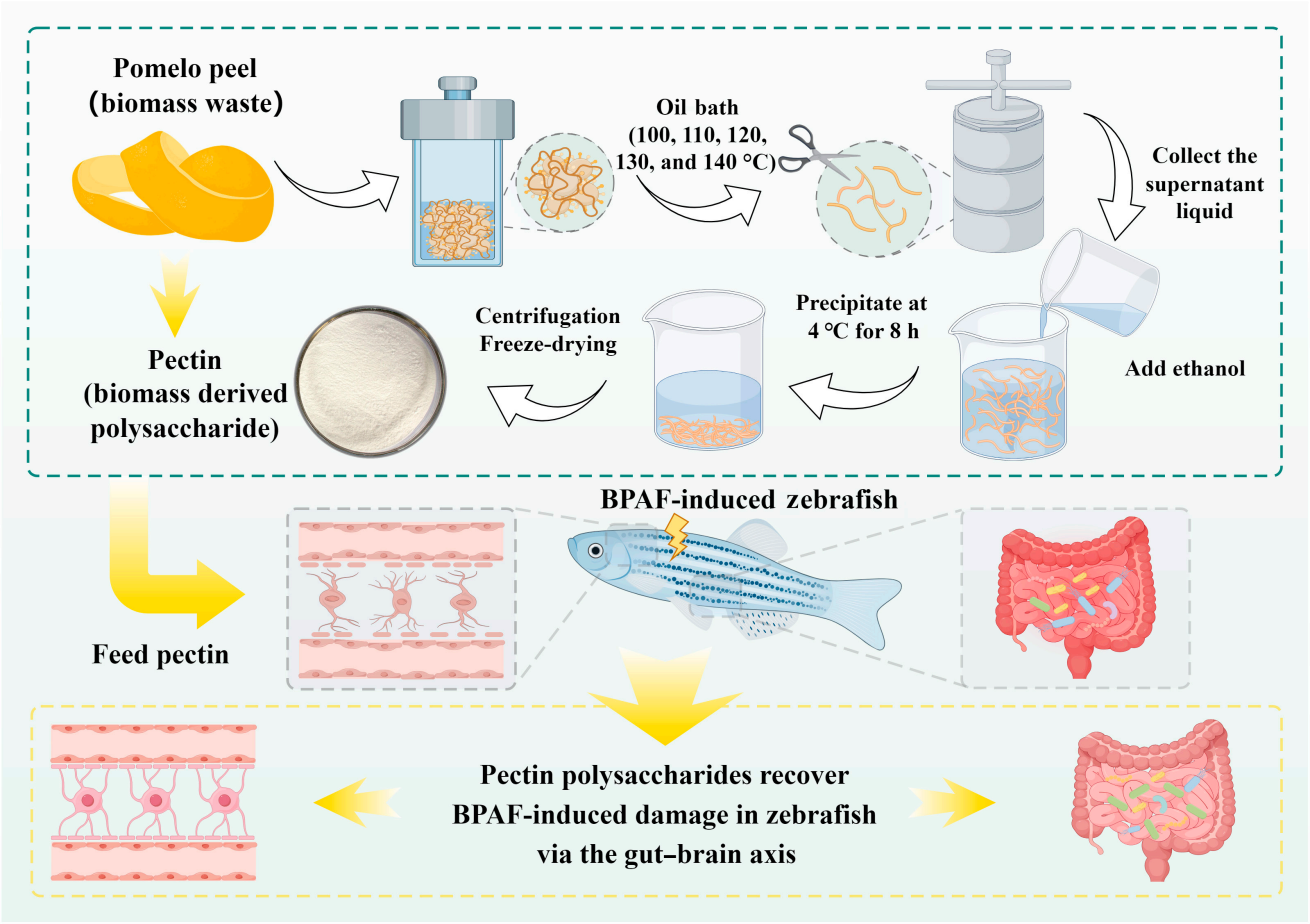
Schematic diagram of biomass pectin preparation and its functional properties (by Figdraw; export ID: APYWU7aa8e).

The pectin samples were prepared following the procedure outlined in Fig. 1. The glycosyl composition and molecular weight of the pectins extracted at different temperatures are summarized in Table S1. The results indicated the main constituent monosaccharides were arabinose (Ara), galactose (Gal), glucose (Glc), xylose (Xyl), mannose (Man), and galacturonic acid (GalA). With increasing extraction temperature, the contents of Ara, Xyl, and Man in the resulting pectins decreased. The GalA content reached a maximum of 39.32% at 120 °C, suggesting that moderate heat exposure facilitated loosening of the cell wall and promoted the release of homogalacturonan (HG) regions. However, the GalA content declined at temperatures above 130 °C, indicating that excessive heat caused backbone cleavage and degradation of HG segments into oligo- or monosaccharides [20]. The Gal content exhibited variations, rising markedly to 33.05% at 140 °C, whereas the Glc content decreased significantly, likely due to the elimination of hemicellulose or starch residues [21].

Gel permeation chromatography (GPC) analysis revealed that the weight-average molecular weight (Mw) increased with temperature up to 130 °C, reaching a maximum of 8.45 × 10^4^ g/mol, and then decreased to 4.67 × 10^4^ g/mol at 140 °C. The number-average molecular weight (Mn) showed a similar trend, whereas the polydispersity index (PDI) increased with temperature, indicating reduced molecular uniformity at elevated temperatures. These variations suggest that pretreatment at 120 to 130 °C favors the production of pectin with a higher GalA content and a larger molecular size, both of which contribute to enhanced gelation and solution viscosity [22]. Beyond these physicochemical attributes, the GalA backbone and the Gal-rich side chains are critical determinants of pectin’s bioactivity. GalA content not only maintains structural integrity but also facilitates crucial interactions with immune receptors and gut microbiota [23]. Similarly, Gal residues function as key recognition motifs for biological interactions and provide fermentable substrates that promote the growth of beneficial bacteria, ultimately contributing to the observed neuroprotective and microbiota-modulating effects [23].

### FT-IR and XPS analysis of obtained pectins

Fourier transform infrared (FT-IR) spectroscopy was employed to examine functional group differences among pectins obtained under distinct extraction conditions (Fig. S1a). The strong absorption peak at 3,205 cm^−1^ corresponds to O–H stretching vibrations stemming from intra- and intermolecular hydrogen bonding. The band at 2,930 cm^−1^ is attributed to the stretching and bending vibrations of C–H in –CH_2_ groups. The peak at 1,740 cm^−1^ is characteristic of esterified carboxyl groups, whereas the band at 1,427 cm^−1^ corresponds to weak C–H bending vibrations, representing symmetric deformation of CH_2_ and C–OH groups [24]. The absorption at 891 cm^−1^ indicates the presence of β-glycosidic linkages, whereas the peak near 830 cm^−1^ is indicative of α-glycosidic linkages. Additionally, the broad band between 875 and 560 cm^−1^ reflects characteristic carbohydrate structural features [25].

X-ray photoelectron spectroscopy (XPS) was further used to analyze the functional group composition of the pectins. The C (1s) peaks in the spectra were assigned to 284.6 eV for aromatic and substituted aliphatic carbons (C–C and C–H); 286.6 eV for phenolic carbons, ether carbons, or carbon–sulfur bonds (C–O–C, C–OH, and C–S); and 289.0 eV for carboxyl groups (O–C–O or C=O) (Fig. S2 and Table S2) [25]. The response values for these carbon structures are summarized in Fig. S1b. The relative content of carbon species increased for pectins extracted at 100 to 120 °C and then decreased for those extracted at 120 to 140 °C. Notably, the relative abundance of C–OH reached its maximum at 120 °C, with a response value of 3,374.33 a.u. This peak suggests that hydroxyl group content, which is closely associated with pectin’s bioactivity, varies with extraction conditions and may consequently influence the biological properties of the resulting pectins [26].

### Evaluation of the biosafety and antioxidant activity of pectin

**Fig. 2.**
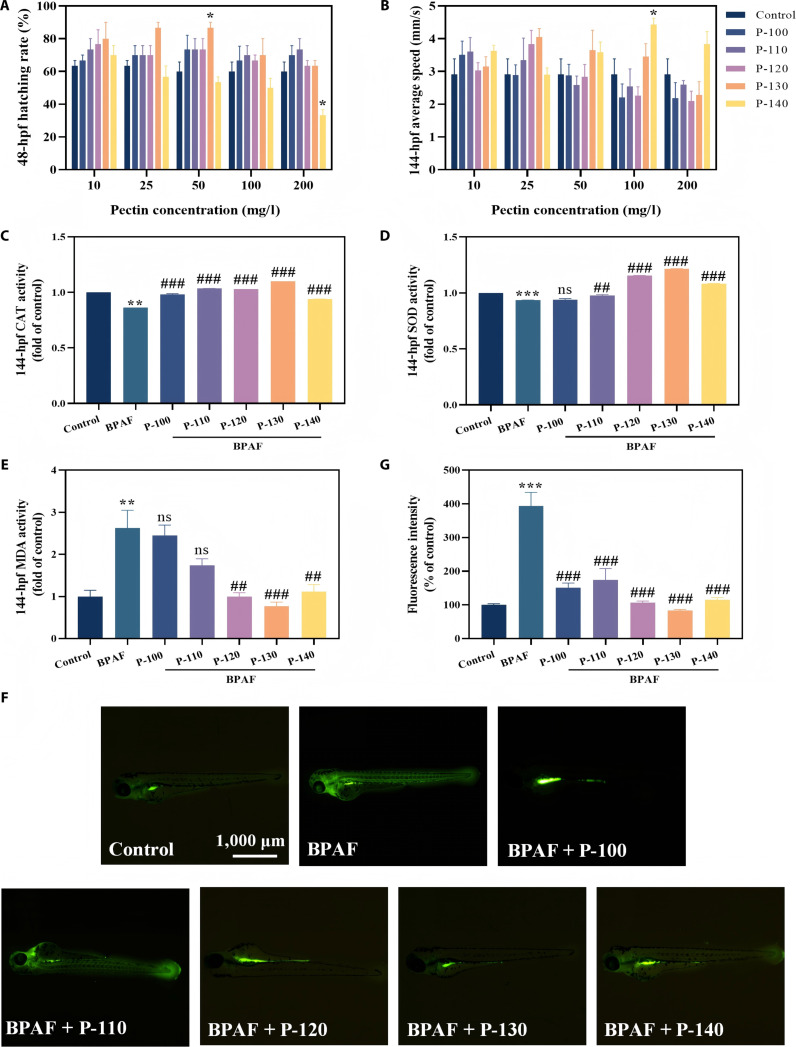
Effects of different pectin samples on the 48 h postfertilization (hpf) hatching rate (A); average speed at 144 hpf (B); oxidative-stress-related indicators at 144 hpf: catalase (CAT) (C), superoxide dismutase (SOD) (D), and malondialdehyde (MDA) (E); reactive oxygen species (ROS) staining images (F); and ROS fluorescence intensity (G) of zebrafish (*P < 0.05, **P < 0.01, ***P < 0.001, ^#^*P* < 0.05, ^##^*P* < 0.01, and ^###^*P* < 0.001; *P is compared with the control group, and ^#^*P* is compared with the bisphenol AF [BPAF] group).

The hatching rate at 48 h postfertilization (hpf) and motor behavior at 144 hpf in zebrafish were assessed to evaluate the biocompatibility of the different pectin samples, as shown in Fig. 2A and B. All pectin-treated groups (10 mg/l) exhibited slightly higher hatching rates than the control. At 25 mg/l, hatching rates were comparable to those of the control, indicating minimal embryonic toxicity consistent with the known biosafety of natural polysaccharide-derived polymers [27]. A slight reduction in hatching rate was observed for P-140 at 50 mg/l, although without evident toxicity. Concurrently, a modest decrease in swimming velocity occurred (P-110 decreased from 2.91 to 2.59 mm/s), suggesting low-level stress induction at higher concentrations, similar to nonspecific stress responses reported for polysaccharides at elevated doses [28,29]. Hatching rates remained statistically unchanged relative to those of the control at 100 mg/l, while mild toxicity appeared for P-140 at 200 mg/l. Based on combined hatching and behavioral data, 25 mg/l was selected as the safe and effective concentration for subsequent restorative experiments.

The antioxidant activities of various pectin samples were initially evaluated by determining their 2,2-diphenyl-1-picrylhydrazyl (DPPH) scavenging capacity, hydroxyl radical (·OH) scavenging capacity, and hydrogen peroxide (H_2_O_2_) scavenging capacity in vitro. As shown in Fig. S3, the in vitro scavenging rates of DPPH, ·OH, and H_2_O_2_ by pectin increased in a concentration-dependent manner. As illustrated in Fig. S3a, P-130 exhibited the strongest DPPH scavenging activity with a half-maximal inhibitory concentration (IC_50_) of 2.26 mg/ml, followed by P-120 (2.76 mg/ml), P-140 (3.23 mg/ml), and P-110 (4.15 mg/ml), while P-100 did not reach its IC_50_ at concentrations below 5 mg/ml. For ·OH scavenging (Fig. S3b), only P-130 achieved an IC_50_ of 4.26 mg/ml within 5 mg/ml. In terms of H_2_O_2_ scavenging (Fig. S3c), P-130 remained the most effective with an IC_50_ of 2.42 mg/ml, followed by P-120 (2.90 mg/ml) and P-140 (2.99 mg/ml), while P-110 and P-100 showed IC_50_ values of 3.46 and 3.83 mg/ml, respectively. Therefore, the pectin extracted at different temperatures exhibits favorable in vitro antioxidant activity, among which P-130 shows the best effect. A zebrafish model of neuro- and cardiotoxicity induced by 0.3 mg/l BPAF was used to evaluate the in vivo antioxidant properties of pectin. As shown in Fig. 2C and D, BPAF exposure significantly suppressed superoxide dismutase (SOD) and catalase (CAT) activities, reflecting impaired antioxidant defense, consistent with oxidative-stress-mediated toxicity reported for BPAF and related contaminants [30]. However, treatment with pectins restored both SOD and CAT activities in BPAF-exposed zebrafish, with P-130 exhibiting the strongest effect. BPAF also markedly increased malondialdehyde (MDA) levels (Fig. 2E), indicating lipid peroxidation and membrane damage. Pectin treatment significantly reduced MDA content, with P-130 again demonstrating the greatest reduction, consistent with its enhanced antioxidative capability. This finding aligns with reports indicating that polysaccharide antioxidants attenuate lipid peroxidation [31]. Based on the characterization data, –OH content does not play a dominant role in determining antioxidant properties. Instead, Gal, GalA, and Mw appear to be the key variables influencing pectin’s antioxidant activity [32].

As shown in Fig. 2F and G, in vivo reactive oxygen species (ROS) fluorescence intensity in BPAF-exposed zebrafish increased to 394.21%, with fluorescence localized predominantly in the head and heart regions. However, pectin treatment markedly attenuated ROS levels in BPAF-exposed zebrafish. In particular, P-130 reduced fluorescence intensity to levels closest to normal (83.09%), demonstrating its superior free radical scavenging efficacy. Collectively, pomelo peel pectin exhibited favorable biocompatibility and pronounced in vivo antioxidant protection in zebrafish. Notably, P-130 showed the strongest efficacy, likely attributable to advantageous molecular structural features (Gal, GalA, and Mw) that confer enhanced bioactivity and antioxidant potency [32]. Higher contents of Gal and GalA suggest more abundant rhamnogalacturonan I side chains and a more intact HG backbone, which provide the molecular basis for interacting with intestinal flora and exerting anti-inflammatory activity [23]. At the same time, a higher Mw facilitates the formation of a more stable 3-dimensional network structure, enhancing its retention capacity and physical barrier function in the intestine [33]. The synergistic effect of these structural characteristics collectively enhances its restorative efficacy.

### Effects of pectin on embryonic movement, survival rate, hatching rate, and heart rate in BPAF-exposed zebrafish

**Fig. 3.**
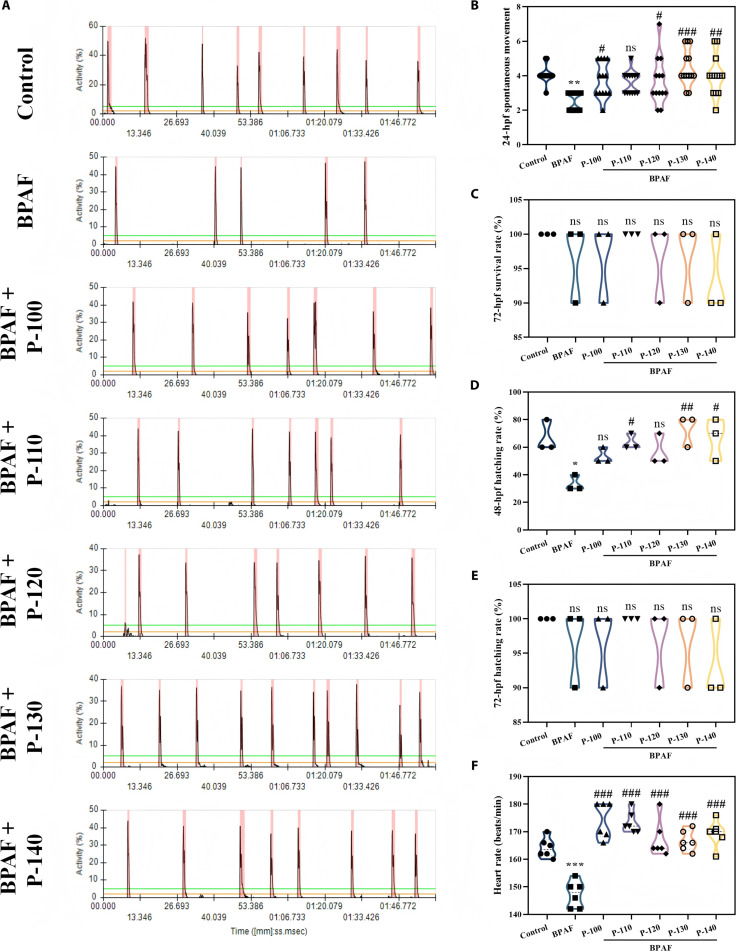
The recovery effects of different pectin samples on the early toxicity induced by bisphenol AF (BPAF) in zebrafish: (A) fetal activity map, (B) fetal movement statistical chart, (C) 72 h postfertilization (hpf) survival rate, (D) 48-hpf hatching rate, (E) 72-hpf hatching rate, and (F) 72-hpf heart rate (*P < 0.05, **P < 0.01, ***P < 0.001, ^#^*P* < 0.05, ^##^*P* < 0.01, and ^###^*P* < 0.001; *P is compared with the control group, and ^#^*P* is compared with the BPAF group).

Embryonic movement, survival rate, hatching rate, and heart rate are key indicators for evaluating early zebrafish development. Therefore, these parameters were examined in BPAF-exposed zebrafish following cotreatment with pectin to assess its restorative effects on BPAF-induced developmental impairment (Fig. 3).

Analysis of embryonic movement vigor (Fig. 3A) and movement counts (Fig. 3B) revealed that the BPAF-induced reduction in embryonic movement was ameliorated by pectin treatment. The P-130 group demonstrated the most effective restoration, returning movement counts to levels comparable to those of the control group. Cotreatment with pectin and BPAF had no significant impact on survival rate at 72 hpf (Fig. 3C), indicating that pectin exhibited no detectable toxicity under these conditions. As shown in Fig. 3D, BPAF exposure significantly delayed hatching at 48 hpf compared to the control. However, treatment with pectins significantly restored the hatching rate in BPAF-exposed embryos, with the P-110, P-130, and P-140 groups exhibiting pronounced effects, increasing hatching by 47.37%, 54.55%, and 50.00%, respectively, relative to that of the BPAF group. Furthermore, hatching rates in both the BPAF and BPAF + pectin groups recovered to normal levels at 72 hpf (Fig. 3E), confirming the absence of toxicity affecting hatching at this developmental stage. Hence, these findings indicate that the ability of pectin to restore hatching in BPAF-exposed zebrafish is primarily reflected in its capacity to recover embryonic activity at 24 hpf. Additionally, the developmental indicator of heart rate was assessed following BPAF exposure (Fig. 3F). The heart rate of control zebrafish was 164 beats per minute (bpm), which decreased significantly to 147 bpm after BPAF induction. Pectin treatment restored heart rates to near-normal levels, with values of 174 bpm (P-100), 173 bpm (P-110), 167 bpm (P-120), 166 bpm (P-130), and 169 bpm (P-140). These results demonstrate the restorative capacity of pectin on BPAF-induced bradycardia, with the P-130 group exhibiting the most effective recovery.

The aforementioned results indicate that pectins extracted at different temperatures exerted varying degrees of restorative effects on embryonic movement, survival rate, and heart rate in BPAF-exposed zebrafish. The P-130 sample consistently demonstrated superior restorative efficacy compared to the other pectins, attributable to its relatively higher Gal content (27.56%), GalA content (37.27%), and Mw compared to those of pectins extracted at other temperatures. BPAF exposure in zebrafish has been demonstrated to induce excessive ROS generation, leading to oxidative stress and subsequent impairment of early development [30]. Notably, the adverse effects of BPAF-induced oxidative stress on zebrafish neural and vascular development can be reversed by antioxidant treatments, including ellagic acid, selenoprotein, and morin [27,28,34]. Therefore, the superior restorative effect of the P-130 sample on BPAF-induced developmental impairments is likely mediated by its potent antioxidant capacity.

### Effects of pectin on fundamental phenotypes in BPAF-exposed zebrafish

**Fig. 4.**
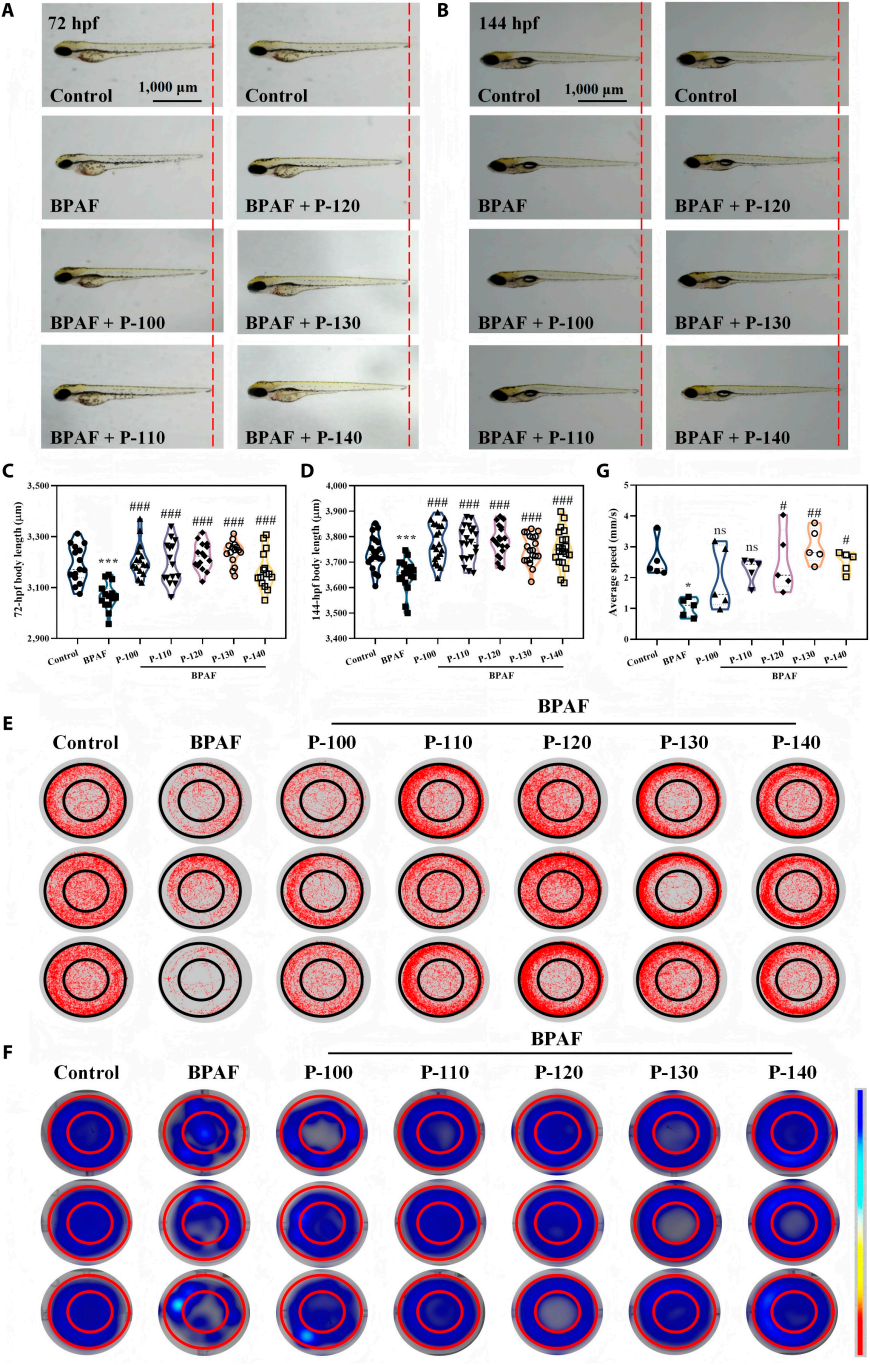
Effects of different pectin samples on the body length and neurobehavioral alteration in zebrafish induced by bisphenol AF (BPAF): (A) typical map of 72 h postfertilization (hpf) body length, (B) typical map of 144-hpf body length, (C) statistical map of 72-hpf body length, (D) statistical map of 144-hpf body length, (E) 144-hpf representative locomotion tracks, (F) 144-hpf track heatmap, and (G) 144-hpf average travel speed (*P < 0.05, **P < 0.01, ***P < 0.001, ^#^*P* < 0.05, ^##^*P* < 0.01, and ^###^*P* < 0.001; *P is compared with the control group, and ^#^*P* is compared with the BPAF group).

Body length and locomotor behavior are fundamental indicators for assessing zebrafish growth and development. Hence, these parameters were measured to provide direct evidence for the restorative efficacy of pectin against BPAF-induced damage (Fig. 4). Visual images (Fig. 4A and B) and quantitative data (Fig. 4C and D) revealed that BPAF exposure significantly inhibited body length growth. Specifically, the BPAF group exhibited body lengths of 3,066 and 3,652 μm at 72 and 144 hpf, respectively, which were significantly reduced compared to those of the control group (3,193 and 3,736 μm). However, treatment with various pectins significantly alleviated this BPAF-induced growth suppression, indicating pectin’s potential to promote zebrafish development.

Neurobehavioral indicators are significant markers of neural development and can be used to evaluate the effectiveness of antioxidants in repairing neural damage [30]. Accordingly, the effects of 25 mg/l pectin on the locomotor behavior of 144-hpf zebrafish, as observed via locomotion tracks and heatmaps, are shown in Fig. 4E and F, respectively. These results demonstrated that BPAF significantly impaired neurological function, evidenced by shortened movement paths and prolonged periods of immobility (indicated by cyan-colored zones), reflecting reduced neural activity. Based on the quantitative analysis of swimming speeds (Fig. 4G), pectin treatment markedly improved locomotor behavior in BPAF-exposed zebrafish. Notably, the P-120, P-130, and P-140 groups restored average swimming speeds to 2.52, 3.03, and 2.51 mm/s, achieving 98.05%, 117.90%, and 97.67% of the control group’s speed, respectively.

The aforementioned results on body length and locomotor behavior revealed that the P-130 group exhibited superior restorative efficacy. This effect is attributed to its potent antioxidant capacity, which effectively mitigated BPAF-induced oxidative stress and promoted the recovery of neural development and physiological function [25]. Consequently, P-130 significantly ameliorated zebrafish locomotor behavior and somatic growth. These findings further underscore the pivotal role of the antioxidant activity of pectin in neuroprotection and suggest its potential application against neurotoxicity.

### Restorative effects of pectin on BPAF-induced central nervous system toxicity in zebrafish larvae

**Fig. 5.**
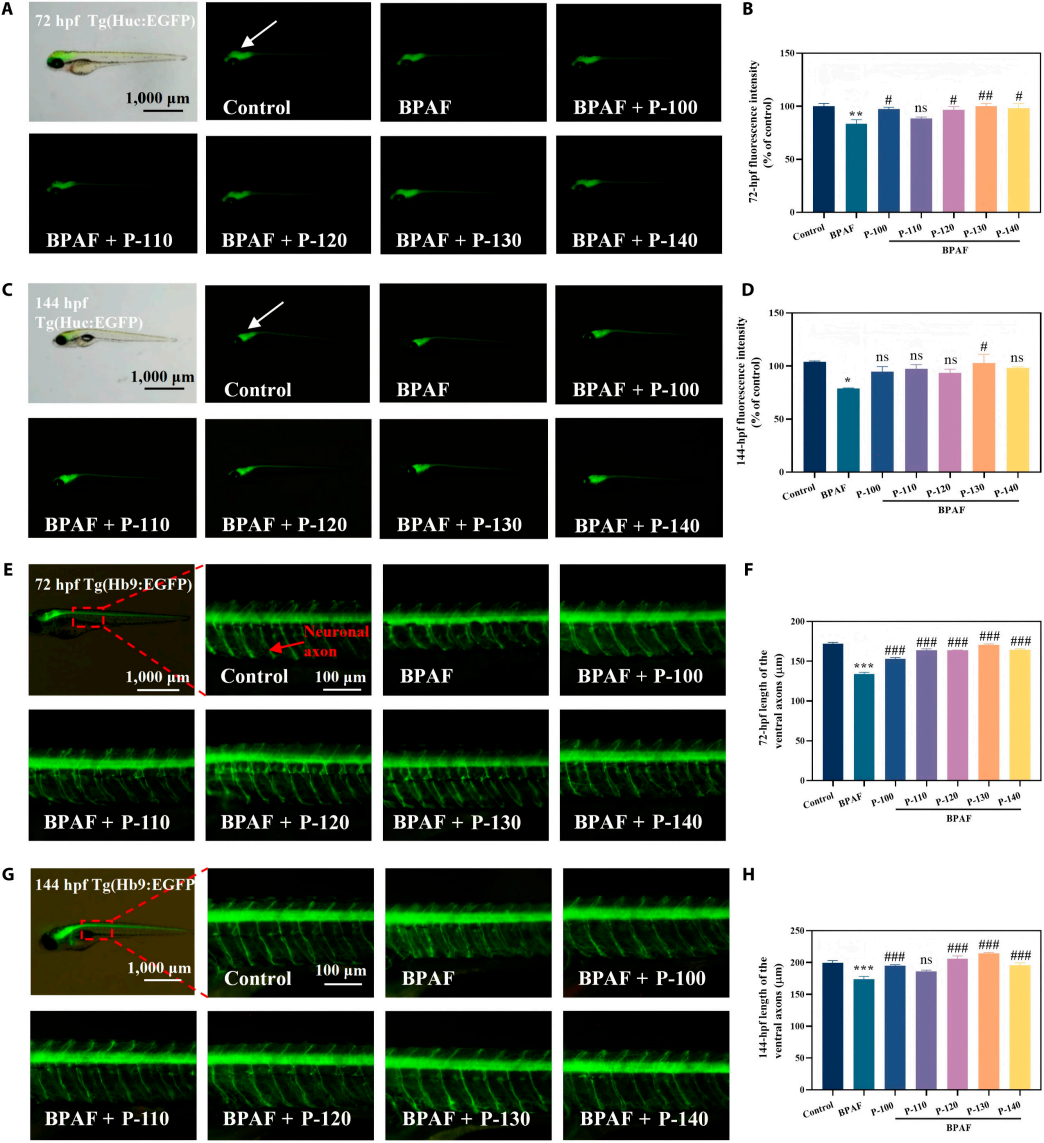
The recovery effects of different pectin samples on bisphenol AF (BPAF)-induced neurotoxicity in the central and motor nerves of zebrafish. Typical images of the central nervous system of zebrafish at 72 h postfertilization (hpf) (A), fluorescence statistics at 72 hpf (B), typical images at 144 hpf (C), and fluorescence statistics at 144 hpf (D). Typical images of the motor nerves of zebrafish at 72 hpf (E), fluorescence statistics at 72 hpf (F), typical images at 144 hpf (G), and fluorescence statistics at 144 hpf (h) (*P < 0.05, **P < 0.01, ***P < 0.001, ^#^*P* < 0.05, ^##^*P* < 0.01, and ^###^*P* < 0.001; *P is compared with the control group, and ^#^*P* is compared with the BPAF group).

A transgenic zebrafish model expressing green fluorescent protein (GFP) is frequently used in zebrafish research to visualize neurodevelopmental abnormalities induced by BPAF and their subsequent repair through antioxidant treatment. In this work, the GFP expression intensity in the brain and spinal cord of Tg(Huc:GFP) zebrafish significantly decreased to 83.56% of control levels following BPAF exposure at 72 hpf. Pectin treatment restored expression to 88.54% to 100.05% compared to the control group, with the P-130 group achieving 100.05% (Fig. 5A and B). For the Tg(Huc:GFP) zebrafish at 144 hpf (Fig. 5C and D), only the P-130 group exhibited significant restoration, reaching 102.56%, thereby demonstrating its superior capacity to repair central neuronal damage. Similarly, quantification of motor neuron axonal length in the Tg(Hb9:GFP) model (Fig. 5E to H revealed that BPAF significantly reduced axonal length to 77.91% and 87.04% of that of control at 72 and 144 hpf, respectively. Pectin treatment restored axonal lengths to 88.94% to 99.09% and 93.12% to 107.28% compared to those of the control group at 72 and 144 hpf, with the P-130 group exhibiting optimal recovery at 99.09% and 107.28%, respectively. These results further corroborate the aforementioned findings: pectin P-130, characterized by higher Gal and GalA contents alongside a greater molecular weight, displays the strongest antioxidant activity and provides superior restorative effects against BPAF-induced neuronal damage. The insignificant differences in effects among different samples for certain measured indicators are closely related to the high similarity in their core structural characteristics, such as similar molecular weight ranges and consistent monosaccharide composition ratios.

To investigate the underlying mechanisms, the expression of key genes involved in neurodevelopment and oxidative stress response (*neuroD*, *α1-tubulin*, *gfap*, *syn2a*, *shha*, and *gap43*) was assessed under BPAF induction (Fig. S4). Pectin treatment, particularly with P-130, significantly up-regulated the expression of these genes: *neuroD* (227.21%), *α1-tubulin* (147.00%), *gfap* (264.66%), *syn2a* (336.25%), and *gap43* (264.07%) relative to control levels. This indicates that pectin extracted at 130 °C effectively promotes the repair of BPAF-induced neural damage by enhancing the expression of neurodevelopment-associated genes. Its superior performance is likely intrinsically linked to its specific monosaccharide composition, in which GalA functions as a multielectron donor to enhance antioxidant activity [35]. Additionally, polysaccharides containing Ara and Gal derived from mulberry fruits have been proven to possess potent antioxidant activity, which also indicates that monosaccharide composition is a key factor for the antioxidant activity of pectin [36].

**Fig. 6.**
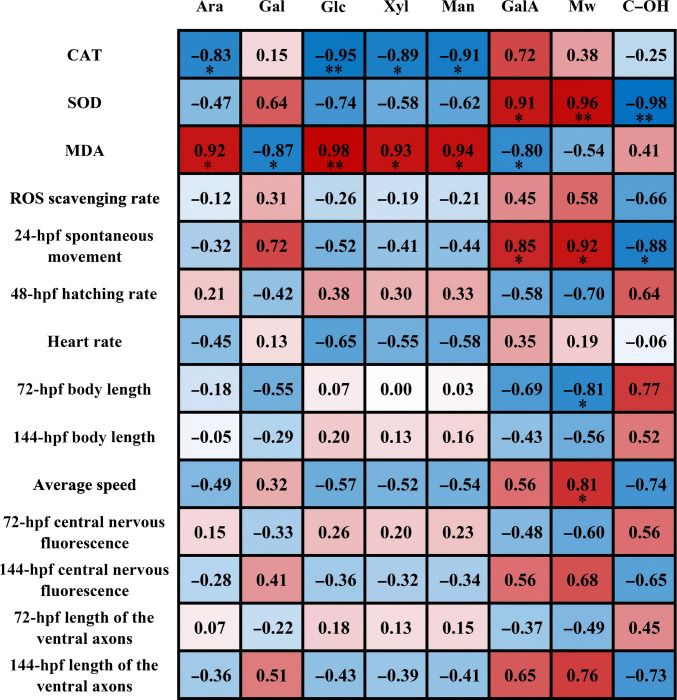
Heatmap depicting the structure–activity relationship of pectin.

Heatmap analysis (Fig. 6) identified key correlations between pectin physicochemical properties and bioactivity in BPAF-exposed zebrafish. Ara, Glc, Xyl, and Man levels were negatively correlated with CAT activity but positively correlated with MDA content, with Glc showing the strongest significance in both relationships. GalA and Mw were positively correlated with SOD activity, whereas C–OH content was negatively correlated with both SOD activity and embryonic movement at 24 hpf. In addition, Mw showed a negative correlation with body length at 72 hpf but a positive correlation with average swimming speed. These results suggest that monosaccharide composition, particularly Glc content, may influence the antioxidant defense system, potentially by exacerbating oxidative stress or via BPAF-induced metabolic effects. Moreover, high-Mw pectins enriched in GalA appear to mitigate oxidative and developmental damage, supporting further investigation into their structure-specific protective mechanisms [37]. P-130 was confirmed as the most effective fraction for alleviating oxidative stress and developmental damage, which is a comprehensive result of its structural characteristics rather than a single factor. Although the contents of Glc and GalA in P-130 were not higher than those in other samples, its relatively high Mw was positively and significantly correlated with SOD activity. This structural characteristic is likely an important contributor to the antioxidative and protective effects of P-130 and may partly explain its superior activity compared with other fractions.

### Effects of pectin on body length, body weight, and behavioral traits in BPAF-exposed adult zebrafish

**Fig. 7.**
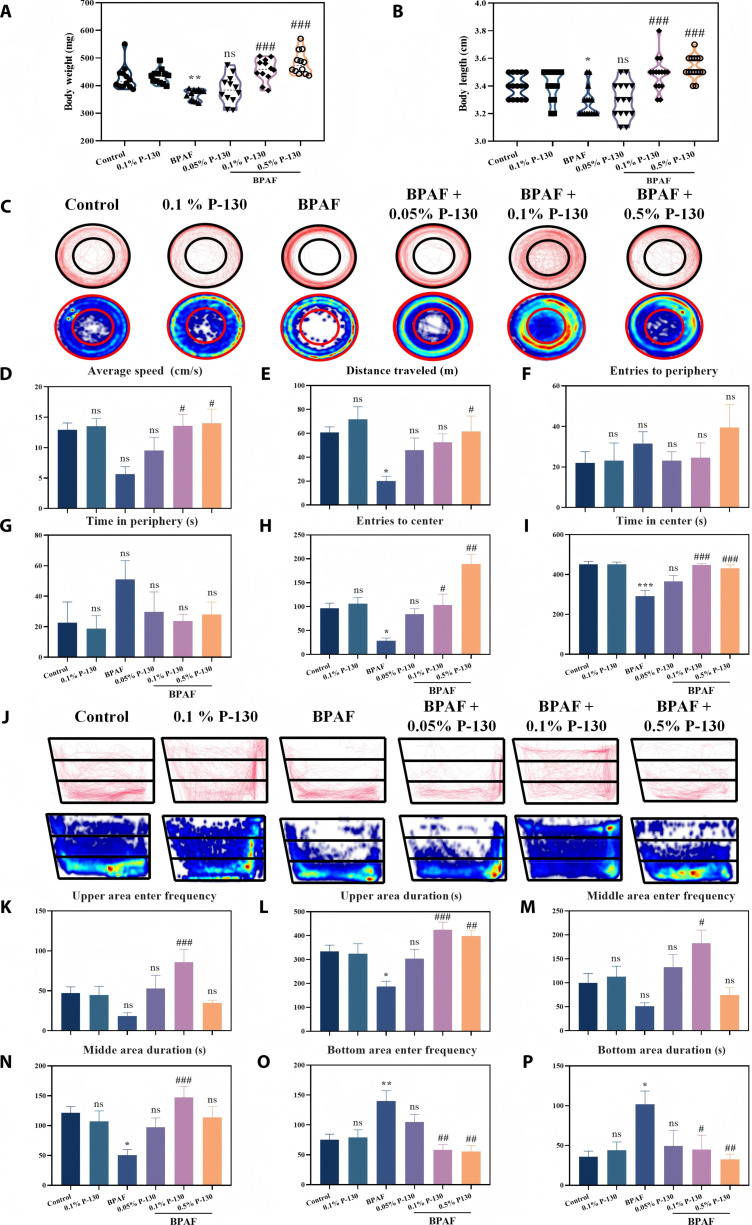
Effects of different treatments on the body weight (A) and body length (B) of adult zebrafish and their anxiety behaviors. The open-field test: (C) representative locomotion tracks and heatmap, (D) average speed, (E) total distance, (F) frequency of entering the fringe, (G) duration in the fringe, (H) frequency of access to the central area, and (I) duration in the central area. The novel tank diving test: (J) track map and heatmap, (K) frequency at the top, (L) duration at the top, (M) frequency at the middle, (N) duration at the middle, (O) frequency at the bottom, and (P) duration at the bottom (*P < 0.05, **P < 0.01, ***P < 0.001, ^#^*P* < 0.05, ^##^*P* < 0.01, and ^###^*P* < 0.001; *P is compared with the control group, and ^#^*P* is compared with the bisphenol AF [BPAF] group).

The adult zebrafish experiment further verified the effectiveness of the optimal sample P-130. Based on the established biosafety of pectin in larval zebrafish, this study used adult zebrafish to examine the restorative effects of different pectin concentrations (0.05%, 0.1%, and 0.5%) against BPAF-induced growth retardation (Fig. 7A and B). Dietary supplementation with 0.1% and 0.5% pectin significantly restored body growth, as evidenced by increases in both body weight (up to 31.34%) and body length (up to 7.7%) compared to those in the group with BPAF-induced injury.

Anxiety-like behavior, a core neurobehavioral phenotype, was assessed using the open-field test (Fig. 7C to I). BPAF exposure significantly reduced the time that adult zebrafish spent in the central zone and increased the time spent in the peripheral zone during open-field swimming. In contrast, treatment of BPAF-exposed zebrafish with 0.1% P-130 restored the frequency of entries into the central zone to levels comparable to those of the control group (103.13%), demonstrating its efficacy in alleviating BPAF-induced anxiety-like behavior. Notably, treatment with 0.1% P-130 alone did not affect anxiety behavior relative to the control, indicating that pectin is safe at this concentration.

The restorative effect of pectin was further examined using the novel tank diving test, a standard behavioral paradigm based on conserved neural circuits regulating anxiety. The corresponding results are presented in Fig. 7J to P. BPAF exposure caused adult zebrafish to spend more time in the lower tank area and less time in the upper and middle areas, consistent with anxiety-like responses [38]. Conversely, the BPAF with 0.1% P-130 group exhibited behavior comparable to that of the control (Fig. 7J), indicating no anxiety induction and highlighting the ability of P-130 to attenuate BPAF-induced anxiety. Further analysis of zebrafish frequency and duration in different tank regions (Fig. 7K to P) revealed that pectin treatment increased both the frequency and duration of entries across all zones. Most notably, the BPAF with 0.1% P-130 group exhibited entry frequencies into the top, middle, and bottom zones reaching 181.24%, 182.82%, and 78.81% of control levels, respectively, with corresponding durations at 127.57%, 121.15%, and 121.16% of that of control. These results further confirm the anxiolytic effect of P-130 at a concentration of 0.1%, which effectively restored anxiety-like behavior in the open-field test. The reduced restorative effect of 0.5% P-130 pectin on anxiety-like behavior in zebrafish compared to that of the 0.1% concentration may be attributed to the excessive adsorption of substances critical for cellular function by pectin at high concentrations. This leads to decreased bioavailability within the system, indirectly causing inhibitory or toxic effects [39]. Additionally, while an appropriate amount of pectin can promote the growth of probiotics in the gut, excessive pectin may interfere with nutrient absorption and intestinal motility due to overfermentation and gas production [40]. Collectively, these findings suggest that the altered behavioral phenotypes are associated with the modified expression of genes regulating second messenger pathways and key neurochemicals [41], indicating that pectin may exert its effects through neuromodulatory pathways.

### Effects of pectin on the brain of adult zebrafish

**Fig. 8.**
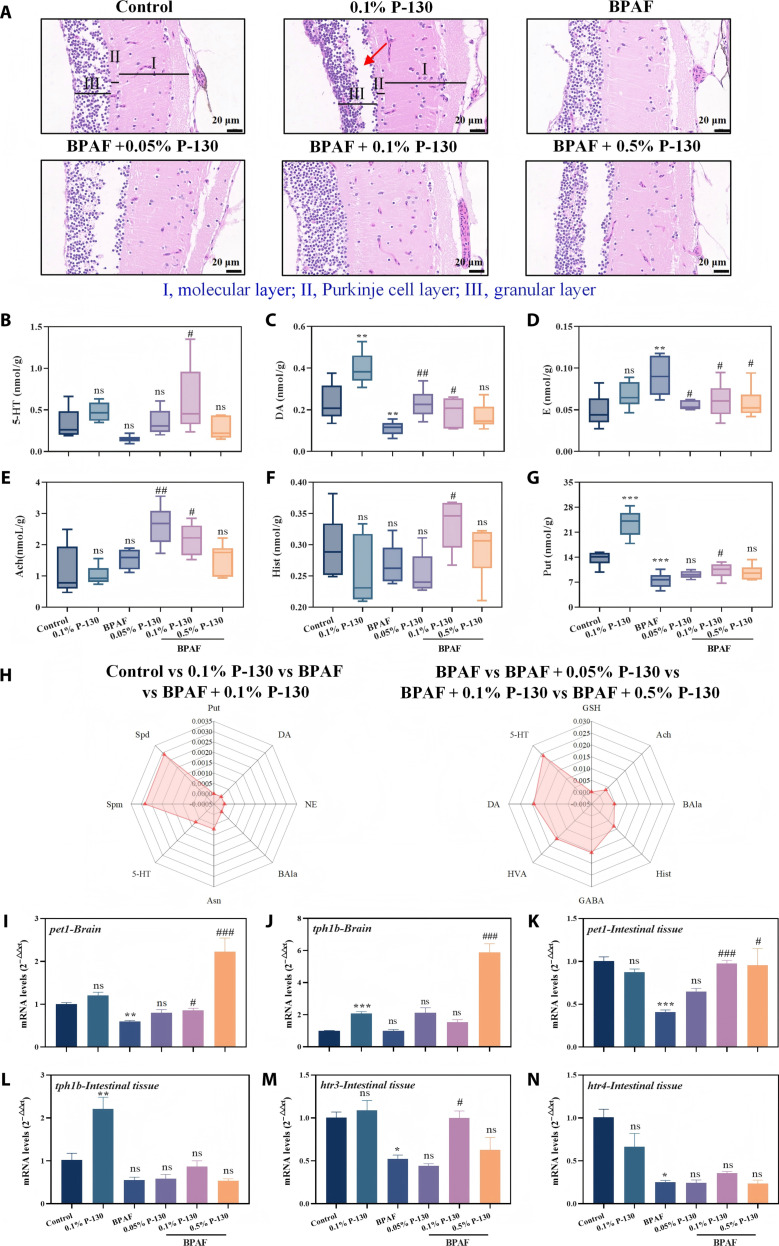
Effects of different treatments on zebrafish brain tissue, brain neurotransmitter content, neurotransmitter radar charts, brain neurotransmitter-related gene expression, and intestinal neurotransmitter-related gene expression: (A) brain section image, (B) 5-hydroxytryptamine (5-HT), (C) dopamine (DA), (D) epinephrine (E), (E) acetylcholine (Ach), (F) histamine (Hist), (G) putrescine (Put), (H) radar map, (I) *pet1-Brain*, (J) *tph1b-Brain*, (K) *pet1-Intestinal tissue*, (L) *tph1b-Intestinal tissue*, (M) *htr3-Intestinal tissue*, and (N) *htr4-Intestinal tissue* (*P < 0.05, **P < 0.01, ***P < 0.001, ^#^*P* < 0.05, ^##^*P* < 0.01, and ^###^*P* < 0.001; *P is compared with the control group, and ^#^*P* is compared with the bisphenol AF [BPAF] group).

Typically, the cerebellum is critically involved in somatic balance and motor coordination. To further elucidate underlying mechanisms, histological analysis of brain tissue, quantification of brain neurotransmitter levels, and assessment of brain- and gut-related gene expression were performed (Fig. 8). Figure 8A shows that control zebrafish exhibited well-defined cerebellar architecture with distinct molecular, Purkinje cell, and granular layers. In contrast, BPAF exposure produced a markedly obscured trilaminar architecture, characterized by a near absence of the Purkinje cell layer, substantial reduction and partial disintegration of granular cells, and displaced and diminished nuclei within the molecular layer, features indicative of severe cerebellar damage. These observations align with reports that BPAF induces meningeal thickening, edema, neuronal loss, and impaired motor coordination [42]. Administration of pectin, particularly in the BPAF with 0.1% P-130 group, significantly restored cerebellar structure toward control levels, indicating that pectin protects against BPAF-induced cerebellar injury and supports recovery of motor coordination.

Anxiety-like behaviors are frequently associated with dysregulation of neurotransmitter systems. Figure S5 presents a heatmap of neurotransmitter alterations in zebrafish brains across treatment groups. BPAF markedly suppressed multiple neurotransmitters, whereas the BPAF with 0.1% P-130 group significantly restored neurotransmitter levels toward normal. The brain serotonin (5-hydroxytryptamine [5-HT]) system is well-known to modulate anxiety behavior [43]. In this, BPAF potentially exacerbated anxiety by reducing 5-HT levels in the zebrafish brain to 51.35% of the control levels (Fig. 8B). Pectin treatment restored 5-HT levels to 116.28% (0.05% P-130), 174.47% (0.1% P-130), and 89.66% (0.5% P-130) to those of control, indicating that pectin modulates 5-HT dysregulation and alleviates anxiety in zebrafish. BPAF also reduced DA levels in the zebrafish brain to 47.86% of the control levels (Fig. 8C), consistent with impaired swimming ability and cognitive deficits, whereas pectin restored DA levels in BPAF-exposed zebrafish to 98.33%, 82.03%, and 71.87% of control levels. Norepinephrine increased to 185.63% of control in the BPAF group, whereas pectin restored it to normal levels (113.34% to 129.77%) (Fig. 8D). Neurotransmitter metabolites, including acetylcholine, histamine, and putrescine, exhibited similar regulatory trends (Fig. 8E to G), with the BPAF with 0.1% P-130 group showing the most pronounced restoration.

Radar plot analysis (Fig. 8H) identified 5-HT and DA as the dominant regulatory neurotransmitters, underscoring their pivotal roles in mediating anxiety mitigation. Key genes governing 5-HT synthesis, *pet1* and *tph1b*, were significantly down-regulated upon BPAF exposure but remarkably up-regulated following pectin intervention (Fig. 8I and J), suggesting that pectin exerts neuroprotective effects, at least in part, through modulation of the central 5-HT system. Considering that approximately 95% of endogenously synthesized 5-HT originates from the gastrointestinal tract [44] and that the gut–brain axis serves as a critical bidirectional communication pathway regulating neural function and mood [45], we further assessed the expression profiles of *pet1*, *htr3*, and *htr4* in zebrafish intestinal tissue (Fig. 8K to N). BPAF exposure significantly suppressed the intestinal expression of *pet1*, *htr3*, and *htr4*, whereas treatment with 0.1% P-130 effectively restored *pet1* and *htr3* expression. Notably, *htr3* encodes a ligand-gated ion channel with well-characterized roles in regulating gut motility and visceral sensation [46], and pectin selectively modulated *htr3* without significantly altering *htr4* expression. Collectively, these data demonstrate that BPAF induces coordinated disruption of the 5-HT system across both central and intestinal compartments along the gut–brain axis, while pectin treatment synchronously restores central 5-HT biosynthesis and intestinal 5-HT/*htr3* signaling. This integrated regulation highlights that pectin’s neuroprotective and gut-protective effects are not isolated but mutually interdependent: pectin preferentially modulates *htr3* to improve intestinal function, which in turn modulates central neural activity via the gut–brain axis to alleviate anxiety-like behavior, thereby mediating its comprehensive neuro–gut protective mechanisms against BPAF-induced neurotoxicity.

### Effects of pectin on the intestine of adult zebrafish

**Fig. 9.**
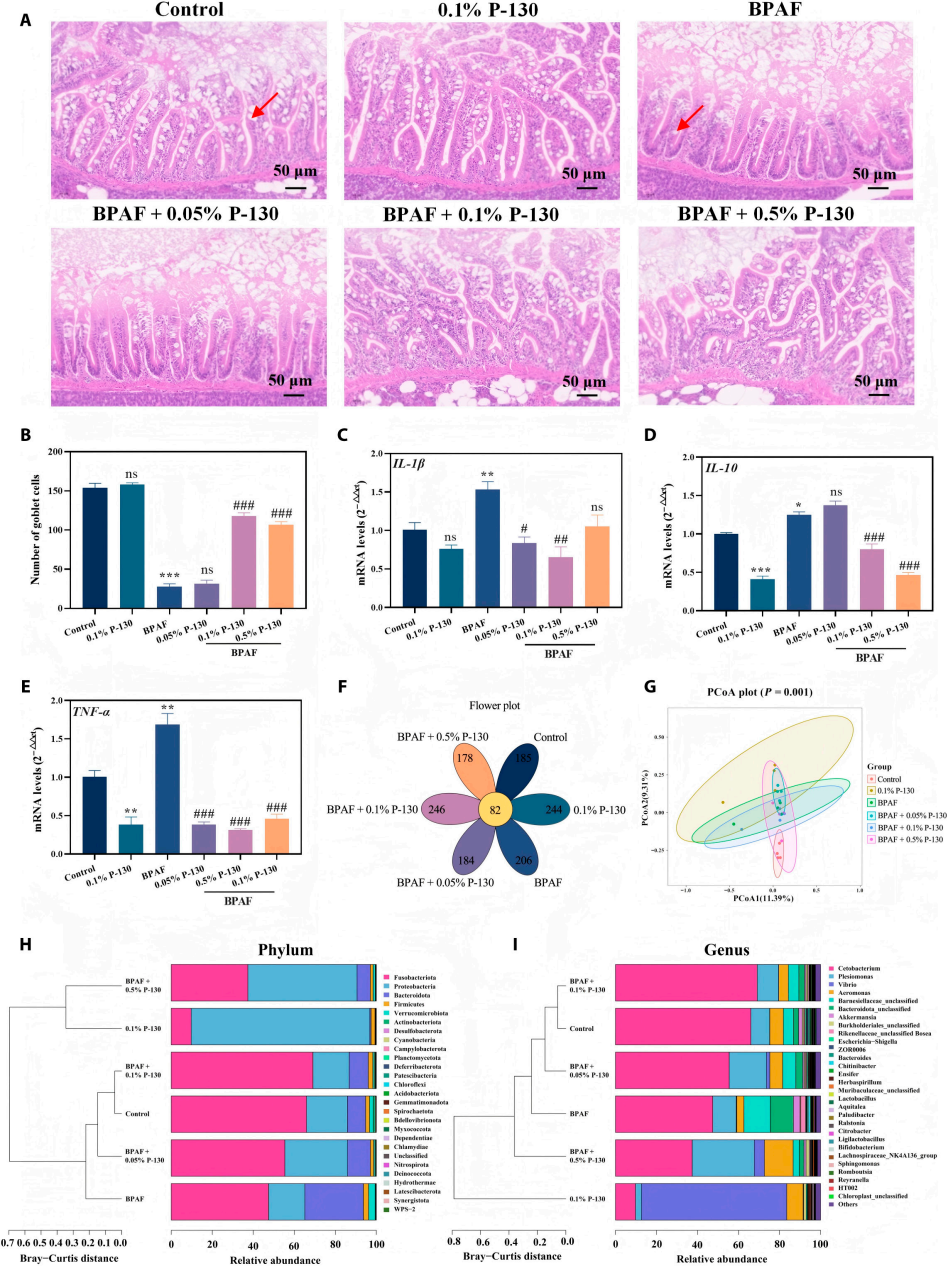
Effects of different treatments on zebrafish intestinal tissue (A), goblet cell number (B), *IL-1β* gene expression (C), *IL-10* gene expression (D), *TNF-α* gene expression (E), Venn diagrams (F), principal coordinates analysis (PCoA) (G), phylum-level accumulation plots (H), and genus-level accumulation plots (I) of the intestinal microbiota in different treatment groups (*P < 0.05, **P < 0.01, ***P < 0.001, ^#^*P* < 0.05, ^##^*P* < 0.01, and ^###^*P* < 0.001; *P is compared with the control group, ^#^*P* is compared with the bisphenol AF [BPAF] group).

Neurotransmitters function as signaling molecules that regulate physiological activities such as movement, mood, and memory, and their production is closely linked to intestinal physiology and the gut microbiota. The intestinal condition of adult zebrafish was histologically evaluated to determine the effects of pomelo peel pectin (Fig. 9). Specifically, paraffin-embedded colon sections were processed for hematoxylin and eosin staining to enable a histopathological assessment of mucosal erosion and ulceration.

Representative images of intestinal tissue and quantitative analysis of goblet cell numbers (Fig. 9A and B) revealed significant morphological differences across treatments. BPAF exposure markedly reduced goblet cell abundance and shortened intestinal villi of zebrafish intestinal tissue. Treatment with 0.1% P-130 and 0.5% P-130 significantly ameliorated these BPAF-induced histopathological alterations and restored goblet cell numbers, with 0.1% P-130 exhibiting superior efficacy. Generally, goblet cells produce Muc2 mucin, which forms the intestinal mucus layer that protects epithelial cells from bacterial invasion [47], while also playing crucial roles in Glc homeostasis and nutrient absorption. Collectively, these findings demonstrate that pectin effectively attenuates BPAF-induced intestinal injury by preserving goblet cell populations and villus architecture, thereby supporting intestinal barrier integrity.

Quantitative real-time polymerase chain reaction (RT-qPCR) analysis of intestinal inflammatory responses (Fig. 9C to E) showed that BPAF significantly up-regulated the expression of interleukin-1β (IL-1β; 151.91%), interleukin-10 (IL-10; 124.83%), and tumor necrosis factor-α (TNF-α; 167.77%) compared to those noted in the control group. Pectin treatment significantly down-regulated the expression of IL-1β (83.71%, 62.20%, and 105.44%), IL-10 (137.41%, 79.99%, and 46.40%), and TNF-α (38.24%, 31.26%, and 46.21%) across the respective concentrations. These findings primarily reflect the transcriptional regulation of inflammatory pathways and suggest that pectin may contribute to the modulation of intestinal immune responses. IL-1β is known to promote intestinal barrier repair and maintenance during stress [48]. Concomitantly, TNF-α is a pleiotropic cytokine with pro-inflammatory and cytotoxic effects, while IL-10 acts as a multifunctional cytokine that limits and resolves inflammatory responses [49]. Overall, interferons, TNF, and interleukins coordinate host defense against pathogens and inflammatory processes. The observed transcriptional changes following pectin treatment indicate a potential role in influencing these signaling networks, although the precise downstream effects on protein abundance and cytokine activity remain to be directly validated.

Given that microbial activity regulates intestinal neurotransmitter synthesis and host–microbiome interactions, the impact of pectin on the gut microbiota was assessed via 16S rDNA amplicon sequencing. Analysis of α-diversity indices (Fig. S6), including observed operational taxonomic units, Chao1, Simpson, Shannon, and Pielou’s evenness, revealed shifts in microbial diversity across groups, indicating that pectin enhances microbial species richness. In addition, 82 core genera shared among all groups were identified in the Venn diagram at the genus level (Fig. 9F). The gut microbiota of zebrafish in each treatment group harbored a distinct set of bacterial species, with counts of 185 (control), 244 (0.1% P-130), 206 (BPAF), 184 (BPAF with 0.05% P-130), 246 (BPAF with 0.1% P-130), and 178 (BPAF with 0.5% P-130). Hence, pectin is a key factor driving divergence in microbial community assembly. β-diversity analysis revealed distinct clustering of gut microbiota among groups (Fig. 9G). A clear separation was observed between the BPAF group and all pectin-treated groups. Notably, the BPAF with 0.5% P-130 group clustered closely with the control and 0.1% P-130 groups, suggesting that this pectin concentration effectively normalized the BPAF-disrupted microbial community.

Analysis at the phylum level (Fig. 9H) showed that *Fusobacteria*, Proteobacteria, and Bacteroidetes were dominant phyla in the zebrafish intestine. Compared to BPAF exposure, the BPAF with 0.1% P-130 and BPAF with 0.5% P-130 groups exhibited reduced *Fusobacteria* abundance (significant for 0.1% P-130) and increased relative abundance of Proteobacteria. The 0.1% P-130 group without BPAF exhibited significantly increased Bacteroidetes abundance. *Fusobacteria* are generally associated with intestinal pathologies such as colorectal cancer and inflammatory bowel disease [50], whereas Bacteroidetes contribute to gut homeostasis and nutrient absorption, possessing broad polysaccharide metabolic capabilities [21]. Notably, as a soluble dietary fiber, pectin serves as a fermentable substrate preferentially utilized by Bacteroidetes, which may explain the selective enrichment of this phylum in the 0.1% P-130 group. In contrast, under BPAF exposure, the lack of a significant increase in Bacteroidetes in the P-130 + BPAF groups may reflect the disruption of normal microbial polysaccharide utilization under environmental stress, further supporting the role of P-130 in selectively modulating beneficial commensals under nonstressed conditions. Hence, it can be speculated that 0.1% P-130 may mitigate inflammatory bowel disease and intestinal infection risks by suppressing detrimental phyla and enriching beneficial ones.

Genus-level analysis (Fig. 9I) showed that *Cetobacterium* was the most dominant genus in the zebrafish intestine. Supplementary data at the genus level are provided in Table S4, and the abundance of *Cetobacterium* is compared in Fig. S7. Compared with the BPAF group, combined treatment with 0.1% P-130 mitigated the BPAF-induced reduction in *Cetobacterium* abundance. *Cetobacterium* is a common commensal involved in vitamin B_12_ synthesis and protein metabolism. However, excessive dominance of a single genus under stress conditions may compromise microbial diversity and intestinal homeostasis. In this context, the moderate recovery of *Cetobacterium* abundance in the P-130 cotreated group likely reflects a rebalancing of the gut microbiota rather than a mere restoration of a single taxon. Such rebalancing is conducive to maintaining microbial diversity and host metabolic function [51], which may collectively contribute to the preservation of intestinal homeostasis and the MGB axis integrity. Collectively, these findings demonstrate that an appropriate concentration of pectin mitigates BPAF-induced intestinal microbial dysbiosis and preserves intestinal homeostasis in zebrafish.

**Fig. 10.**
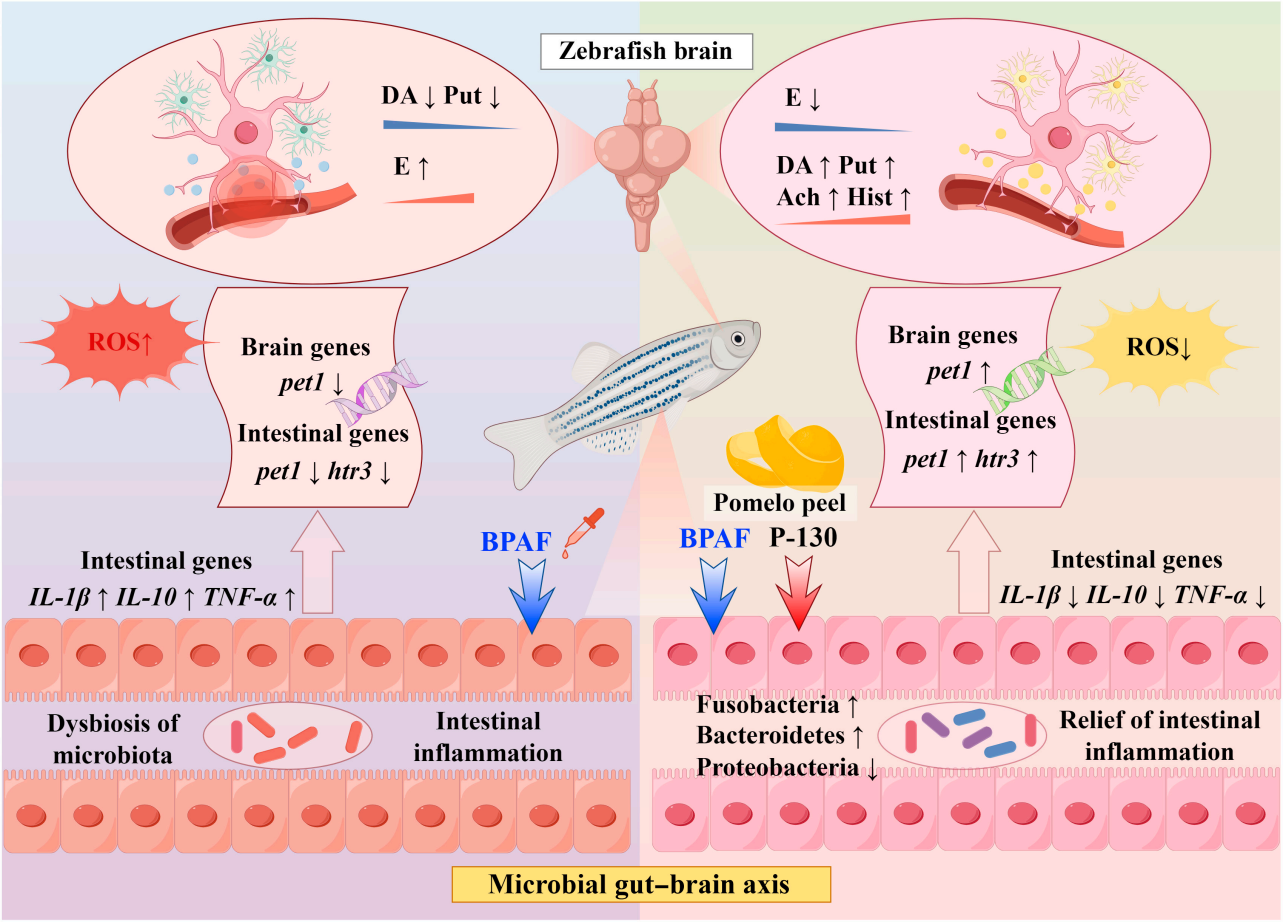
Diagram depicting the mechanism underlying the effect of pectin on bisphenol AF (BPAF)-induced damage in zebrafish through the microbial gut–brain axis (by Figdraw; export ID: YTTIP2246a) (DA, dopamine hydrochloride; Put, putrescine; E, epinephrine; Ach, acetylcholine chloride; Hist, histamine).

Gut dysbiosis is closely associated with neuroinflammation and brain pathology [52]. For example, Bacteroidetes are involved in central nervous system function through their roles in modulating microbial metabolites and participating in neurotransmitter-related pathways, including tryptophan metabolism and serotonin (5-HT) signaling [53]. Therefore, pectin may contribute to maintaining gut microbial homeostasis by influencing the abundance of Bacteroidetes, which is potentially linked to alterations in neurotransmitter dynamics and the observed alleviation of anxiety-like behavior in zebrafish. To address the complexity of these interactions, it should be noted that the relationship among microbiota composition, gut-derived metabolites, gene expression, and brain neurotransmitters represents a coordinated and multifactorial regulatory network rather than a single linear pathway. Based on the integrative analysis of sequencing data from gut microbiota, intestinal metabolites, host gene expression, and brain neurotransmitter levels, the protective effects of pectin P-130 against BPAF-induced damage in zebrafish are summarized and illustrated in Fig. 10. Collectively, these findings suggest that pectin exerts neuro–gut protective effects through interconnected regulatory processes, supporting its potential as a natural agent against environmental toxicant-induced neural injury.

## Conclusion

Pretreatment temperature critically governed the structural and biofunctional properties of pomelo peel pectin. Extraction at 130 °C yielded P-130 pectin with optimal GalA content (37.27%), molecular weight (8.45 × 10^4^ g/mol), and hydroxyl group abundance. In vivo studies demonstrated that P-130 effectively alleviated BPAF-induced toxicity in zebrafish by restoring developmental parameters, reducing oxidative stress, and protecting neurodevelopment. Furthermore, P-130 modulated the gut–brain axis, by restoring neurotransmitter homeostasis (174.47% increase in 5-HT and 98.33% increase in DA), enriching beneficial microbiota (Bacteroidetes and *Cetobacterium*), suppressing inflammation (31.26% reduction in TNF-α), and alleviating anxiety-like behavior. These multifaceted effects, rooted in P-130’s distinct structural attributes, underscore its potential as a natural therapeutic against environmental toxicants through antioxidant, neuroprotective, and microbiota-modulating mechanisms. Future studies should evaluate P-130’s bioavailability, long-term safety, and therapeutic efficacy in mammalian models of environmental toxin exposure, thereby paving the way for its development as a functional food ingredient or nutraceutical.

## Materials and Methods

### Raw materials and reagents

Mature yellow pomelos (*Citrus maxima* cv. Wentan) were purchased from a local fruit market. The pomelo peels were manually separated and rinsed with deionized water to remove surface impurities. The dried samples were ground into fine powder (20 to 80 mesh) using an electric grinder and stored in sealed containers for subsequent use.

All zebrafish larvae and adult zebrafish used in this study were obtained from the Nanjing Institute of Environmental Sciences, Ministry of Ecology and Environment, China. In particular, Tg(Huc:GFP) is a transgenic zebrafish line in which the expression of GFP is driven by the neuron-specific Huc promoter. This strain allows for the visualization of neurons through fluorescence labeling. Tg(Hb9:GFP) is a transgenic zebrafish line in which the neuron-specific promoter hlxb9 (hb9) drives the expression of GFP. It is primarily used to visualize motor neurons and their axons. Assay kits for CAT and SOD, as well as the MDA assay kit, were obtained from Beyotime Biotechnology Co., Ltd. (Shanghai, China). The RNA extraction kit, complementary DNA first-strand synthesis kit, and RT-qPCR kit were all purchased from Nanjing Vazyme Biotechnology Co., Ltd. (Nanjing, China).

### Hydrothermal treatment for obtaining pomelo peel pectin

Pomelo peel powder (1.5 g) was mixed with 75 ml of distilled water (solid–liquid ratio 1:50) in an oil-bath kettle, followed by hydrothermal extraction in an oil-bath pot for 1 h at different temperatures (100, 110, 120, 130, and 140 °C). After extraction, the mixture was filtered through a G3 funnel, and the filtrate was kept cool at room temperature. Subsequently, the pectin was precipitated by adding anhydrous ethanol at a 3:1 (v/v) ratio and refrigerated at 4 °C for 8 h. To separate the suspended clots of pectin from the ethanol, the mixture was centrifuged at 8,000 rpm for 10 min, filtered using filter paper, and washed twice with anhydrous ethanol. The crude polysaccharide was dissolved in water, and an equal volume of 5% trichloroacetic acid was added for protein removal under low-temperature stirring. After centrifugation, the supernatant was dialyzed against ultrapure water using a 200-Da dialysis bag for 72 h to remove trichloroacetic acid. The solution inside the dialysis bag was collected, concentrated by rotary evaporation, and then freeze-dried to obtain polysaccharide powder. The pectin samples extracted at 100, 110, 120, 130, and 140 °C were designated as groups P-100, P-110, P-120, P-130, and P-140, respectively. The pectin powder was stored in a silica gel desiccator until further use.

### Composition and structural analysis of pomelo peel pectin

#### Determination of polysaccharide composition

The polysaccharides in the pectins were back-calculated after acid hydrolysis into monosaccharide with 4% sulfuric acid. The monosaccharide of Ara, Gal, Glc, Xyl, and Man in the acid hydrolysate was determined by high-performance anion-exchange chromatography, which was quantified using a Dionex ICS-5000 system (Thermo Fisher Scientific Inc., Waltham, USA), following the procedure described by Yan et al. [25]. GalA was quantified using a Dionex ICS-3000 system (Thermo Fisher Scientific Inc., Waltham, USA) according to the method of Wang et al. [54]. All analytical procedures were performed in 3 parallel runs.

#### Determination of molecular weight

The molecular weight distribution of pectin samples was determined by GPC according to the method described by Yan et al. [25]. Mn, Mw, and polydispersity index (PDI = Mw/Mn) were calculated using Agilent GPC software.

#### XPS and FT-IR spectroscopy analysis

For XPS analysis, a small amount of powdered pectin sample was placed in a centrifuge tube, vacuum-dried, and analyzed using an AXIS Ultra DLD X-ray photoelectron spectrometer (Kratos, UK). Full spectra were processed with the XPSPEAK41 software for peak deconvolution and comparison of XPS spectra among different samples. For FT-IR analysis, a small amount of powdered sample was scanned in the wave number range of 4,000 to 500 cm^−1^ [25]. The infrared spectra were analyzed and compared across different pectin samples.

#### In vitro antioxidant activity assay of pectin

Different pectin samples were prepared into solutions with various mass concentrations (0, 1, 2, 3, 4, and 5 mg/ml), and the resulting solutions were stored for subsequent use.

##### Determination of DPPH radical scavenging activity

Two milliliters of polysaccharide solutions at various concentrations was transferred into test tubes, followed by the addition of 2 ml of 0.2 mmol/l DPPH solution prepared with anhydrous ethanol. After mixing, the reaction was carried out in the dark at room temperature for 30 min. The absorbance was measured at 517 nm and recorded as *A*_Sample_. For the control group, 2 ml of polysaccharide samples at various concentrations was added with 2 ml of anhydrous ethanol, and the absorbance at 517 nm was recorded as *A*_Control_. For the blank group, 2 ml of anhydrous ethanol was mixed with 2 ml of DPPH solution, and the absorbance at 517 nm was recorded as *A*_Blank_. Each sample was tested in duplicate. The DPPH radical scavenging activity was calculated using the following formula:

$$\begin{array}{c} \text{DPPH scavenging activity}\;\left(\%\right)=\left(1-\frac{A_{\text{Sample}}-A_{\text{Control}}}{A_{\text{Blank}}}\right)\times 100\% \end{array}$$ (1)

*A*_Sample_ is 2 ml of polysaccharide + 2 ml of DPPH solution, *A*_Control_ is 2 ml of polysaccharide + 2 ml of anhydrous ethanol, and *A*_Blank_ is 2 ml of anhydrous ethanol + 2 ml of DPPH solution.

##### Determination of hydroxyl radical (·OH) scavenging activity

The hydroxyl radical (·OH) scavenging activity was determined using the salicylic acid method. Polysaccharide solutions (1 ml) at various concentrations were transferred into test tubes, followed by the sequential addition of 2 ml of 9 mmol/l FeSO_4_ solution and 2 ml of 9 mmol/l salicylic acid–ethanol solution. The reaction was initiated by adding 2 ml of 8.8 mmol/l H_2_O_2_, followed by incubation in a water bath at 37 °C for 30 min. The absorbance was measured at 510 nm using distilled water as the reference. Considering the inherent absorbance of polysaccharides, a polysaccharide blank was prepared using 2 ml of polysaccharide solution at various concentrations, 2 ml of 9 mmol/l FeSO_4_, 2 ml of 9 mmol/l salicylic acid–ethanol, and 2 ml of distilled water. Each sample was tested in duplicate. The hydroxyl radical scavenging activity was calculated as follows:

$$\begin{array}{c} \cdot \mathrm{OH}\;\text{scavenging activity}\;\left(\%\right)=\left(1-\frac{A_{\text{Sample}}-A_{\text{Control}}}{A_{\text{Blank}}}\right)\times 100\% \end{array}$$ (2)

*A*_Sample_ is 2 ml of polysaccharide + 2 ml of salicylic acid + 2 ml of FeSO_4_ + 2 ml of H_2_O_2_, *A*_Control_ is 2 ml of polysaccharide + 2 ml of salicylic acid + 2 ml of FeSO_4_ + 2 ml of H_2_O, and *A*_Blank_ is 2 ml of H_2_O + 2 ml of salicylic acid + 2 ml of FeSO_4_ + 2 ml of H_2_O_2_.

##### Determination of H_2_O_2_ scavenging activity

One milliliter of polysaccharide solutions at various concentrations was transferred into test tubes, mixed with 6.0 ml of 0.1 mol/l phosphate buffer solution (pH 7.4) and 1.0 ml of 50 mmol/l H_2_O_2_ solution. After shaking and incubating for 10 min, the absorbance was measured at 230 nm using distilled water as the blank control. The H_2_O_2_ scavenging activity was calculated using the following formula:

$$H_{2}O_{2}\;\text{scavenging activity}\;\left(\%\right)=\frac{A_{\text{Control}}-A_{\text{Sample}}}{A_{\text{Control}}}\times 100\%$$ (3)

*A*_Sample_ is 1 ml of polysaccharide + 6 ml of phosphate buffer solution + 1 ml of H_2_O_2_ solution and *A*_Control_ is 1 ml of distilled water + 6 ml of phosphate buffer solution + 1 ml of H_2_O_2_ solution.

### Zebrafish larval experiments

#### Zebrafish maintenance

The zebrafish were maintained in dechlorinated, aerated tap water with specific conditions: temperature set at 28 ± 0.5 °C, dissolved oxygen at 6.0 mg/l, and a pH of 7.3 to 7.5, supported by a continuous water circulation system [55]. A 14-h light/10-h dark photoperiod was applied. Zebrafish were fed 3 times daily at 0830, 1430, and 1830. One-third of the water was replaced daily, and feces and debris were removed to maintain water quality. The rearing facility was isolated from the external environment and disinfected regularly.

Prior to the experiments, adult zebrafish older than 3 months were selected. On the evening before spawning (2030), healthy male and female zebrafish were paired at a 1:1 ratio and placed in transparent spawning boxes with a divider. On the following morning (0800), the divider was removed before turning on the lights. Upon light stimulation, male zebrafish chased and nudged the abdomens of female zebrafish, inducing oviposition. After spawning, the male and female zebrafish were separated and returned to individual tanks for recovery. Fertilized eggs were collected using a Pasteur pipette and rinsed 3 times with aerated water before transferring into glass dishes. Unfertilized or dead embryos were removed 4 to 5 hpf.

#### Biosafety evaluation of pomelo peel pectin in zebrafish larvae

To evaluate the biosafety of pomelo peel pectin extracted at different temperatures, fertilized zebrafish embryos were exposed to pectin solution. Embryo hatching rates at 48 hpf and locomotor behavior at 144 hpf were assessed to evaluate the biosafety of pomelo peel pectins according to the protocol described by Deng et al. [55].

#### Baseline phenotypic analysis of zebrafish larvae exposed to BPAF and pectin

For exposure experiments, BPAF with a concentration of 0.3 mg/l was used to induce neurotoxicity in zebrafish [55]. The zebrafish embryos (30 per group) were continuously treated from 4 to 144 hpf under the following conditions: only BPAF exposure (BPAF group), BPAF exposure combined with pomelo peel pectin (BPAF + pectin group), or untreated controls (control group). During the exposure period, fresh test solutions of the same concentration were renewed daily, and dead larvae were promptly removed. The hatching rate was calculated as the proportion of successfully hatched larvae relative to the total number of embryos, providing a quantitative index of hatching success. Spontaneous embryonic movement was observed using the DanioScope system, which quantifies the number and total duration of embryo movements. This parameter provides a sensitive indicator of chemical interference with early neurodevelopment in zebrafish [34].

#### Evaluation of the antioxidant activity of pomelo peel pectin

The in vivo antioxidant capacity of pomelo peel pectin was evaluated by measuring its ability to scavenge ROS in BPAF-treated zebrafish. Embryos were divided into 3 groups: control, BPAF, and BPAF + pectin cotreatment. Each group was incubated in the corresponding medium for 3 d. Zebrafish larvae were then stained with 2′,7′-dichlorofluorescein diacetate solution at 28 °C for 20 min, followed by 3 washes with phosphate-buffered saline to remove excess dye. Fluorescence intensity was observed under a fluorescence stereomicroscope. In addition, the activities of CAT and SOD and the content of MDA were measured in zebrafish larvae at 144 hpf using bicinchoninic acid assay kits.

#### Behavioral analysis of zebrafish larvae exposed to BPAF and pectin

To evaluate the behavioral effects of pomelo peel pectin in BPAF-exposed zebrafish, 20 larvae at 144 hpf were randomly selected and placed in a 24-well plate. The larvae were transferred to the DanioVision system for observation. After a 10-min acclimation period, free-swimming behavior was recorded for 40 min under alternating light (10 min) and dark (10 min) cycles. Larval locomotor activity and average swimming speed were analyzed using the EthoVision software.

#### Analysis of the neural regenerative effects of pomelo peel pectin in zebrafish larvae

To evaluate the neural regenerative effects of pomelo peel pectin, transgenic zebrafish larvae expressing GFP (Huc-GFP and Tg(Hb9 EGFP)) were used. Larvae were incubated until 72 and 144 hpf, and GFP expression was visualized under a fluorescence microscope. The intensity of GFP fluorescence in Huc-GFP larvae was analyzed to assess central nervous system recovery, while the axonal length of motor neurons in Tg(Hb9:EGFP) larvae was measured to evaluate motor neuron regeneration following BPAF exposure [30].

#### RT-qPCR analysis

Total RNA was extracted from zebrafish samples using a commercial RNA extraction kit, following the manufacturer’s protocol. RNA concentration was measured twice with an OneDrop spectrophotometer, and data were recorded. First-strand complementary DNA synthesis and RT-qPCR were performed using a commercial kit, according to the manufacturer’s instructions. β-Actin was selected as the housekeeping gene, and relative gene expression was calculated using the 2^−ΔΔCt^ method [56]. The sequences of primers used in this study are listed in Table S3.

### Zebrafish adult experiments

#### Experimental design

A total of 360 female adult zebrafish (3 months old, body weight 0.415 ± 0.06 g) were obtained and randomly divided into 6 groups (*n* = 60 per group) of blank control group, model group (BPAF), negative control group (0.1% P-130), and 3 experimental groups (BPAF with 0.05% P-130, BPAF with 0.1% P-130, and BPAF with 0.5% P-130).

Zebrafish in the model and experimental groups were cultured in BPAF solution (50 μg/l). During the experiment, the blank control and model groups were fed with normal *Artemia nauplii*. The negative control group received *Artemia* powder supplemented with 0.1% P-130, while the experimental groups received *Artemia* powder supplemented with 0.05%, 0.1%, and 0.5% P-130, respectively. Feeding was performed 3 times daily (0900, 1400, and 1700) until satiety was achieved. Twelve zebrafish were randomly selected from each group for behavioral assessment in the third week. Fifty zebrafish per group were sacrificed in the fourth week to obtain the brain and intestinal tissues for the analysis of gut microbiota composition and brain neurotransmitter levels.

#### Behavioral assessments of adult zebrafish

The anxiety-like behavior and locomotor activity of zebrafish were assessed following a 4-week exposure period using both the open-field test and the novel tank test. In the open-field test, individual zebrafish were placed in a glass dish (diameter: 10 cm; height: 2 cm) filled with water. The arena was divided into peripheral and central zones. In the novel tank test, each zebrafish was introduced into a trapezoidal tank (height: 15 cm; top length: 28 cm; bottom length: 22 cm; width: 9 cm) filled with water. The tank was virtually divided into 3 horizontal zones of equal size.

In both tests, zebrafish (*n* = 10 per group) were acclimated for 5 min prior to an 8-min behavioral recording session conducted with an OMSO Action 3 camera. After each trial, the apparatus was thoroughly cleaned and refilled with fresh water. The frequency of entries and duration spent in each zone were calculated. All data acquisition and analysis were performed using the EthoVision XT 15 software [55].

#### Histopathological analysis, intestinal 16S rDNA sequencing, and neurotransmitter analysis for adult zebrafish

The brain and intestinal tissues of adult female zebrafish were examined histologically. Whole zebrafish were fixed in 10% buffered formalin, embedded in paraffin, sectioned, and stained with hematoxylin and eosin for pathological evaluation of brain and intestinal morphology [57].

Four zebrafish per group on the fourth week were dissected, and intestinal contents were collected and flash-frozen in liquid nitrogen. Gut microbiota profiling was performed by Shanghai Biotree Biomedical Technology Co., Ltd. (Shanghai, China) using 16S rDNA sequencing. Six zebrafish per group in the fourth week were dissected, and brain tissues were collected and flash-frozen in liquid nitrogen. Neurotransmitter levels in the zebrafish brain were analyzed by Shanghai Biotree Biomedical Technology Co., Ltd. (Shanghai, China).

### Statistical analysis

All experiments were performed in triplicate, and data are expressed as mean ± standard deviation. One-way analysis of variance was performed using SPSS 20.0 and GraphPad Prism software. The statistical significance between groups was assessed, with differences deemed significant at *P and ^#^*P* < 0.05, **P and ^##^*P* < 0.01, and ***P and ^###^*P* < 0.001. An asterisk (*) denotes a comparison with the control group, while a hash (#) signifies a comparison with the BPAF group.

## Ethical Approval

All zebrafish experiments were conducted under ethical approval from the Nanjing Institute of Environmental Sciences (Approval No.: IACUC-20250910).

## Data Availability

All data required to support the conclusions are presented in the main text and the Supplementary Materials.
