# Supplementary material for: Biofunctional Pectin Derived from Pomelo Peel: Structural Insights and Neuro–Gut Protective Mechanisms in Zebrafish under Bisphenol AF-Induced Neurotoxicity
Source: Research (Wash D C). 2026 May 7;9:1263. doi: 10.34133/research.1263 (PMC13150078; doi:10.34133/research.1263)
Supplement: Supplementary 1 — Tables S1 to S4 Figs. S1 to S7 [file research.1263.f1.docx]

**Supporting information**

**Biofunctional Pectin Derived from Pomelo Peel: Structural Insights and Neuro-Gut Protective Mechanisms in Zebrafish under BPAF-Induced Neurotoxicity**

Bowen Yan ^1,3,4^, Junping Deng ^2^, Xinyu Hu ^1,3,4^, Shuo Tang^5^, Qiang Yong^2^, Jie Gu ^6,*^, Caoxing Huang ^2,*^

1. National Key Laboratory for Development and Utilization of Forest Food Resources, Institute of Chemical Industry of Forest Products, Chinese Academy of Forestry, Nanjing 210042, China
2. State Key Laboratory for the Development and Utilization of Forest Food Resources, Nanjing Forestry University, Nanjing 210037, China
3. Key Lab. of Biomass Energy and Material, Jiangsu Province; Key Lab. of Chemical Engineering of Forest Products, National Forestry and Grassland Administration, Nanjing 210042, China
4. International Innovation Center for Forest Chemicals and Materials; Jiangsu Co-Innovation Center of Efficient Processing and Utilization of Forest Resources, Nanjing 210042, China.
5. Nanjing Institute of Comprehensive Utilization of Wild Plants, Nanjing, 211111, China
6. Nanjing Institute of Environmental Sciences, Ministry of Ecology and Environment, Nanjing 210042, China

* Corresponding to: [gujie@nies.org](mailto:gujie@nies.org); hcx@njfu.edu.cn

**I. Supplementary Tables**

**Table S1 Glycosyl composition and molecular weight of** **pectin from pomelo peels.**

| Samples | Ara  （%） | Gal  （%） | Glc  （%） | Xyl  （%） | Man  （%） | GalA（%） | Mw  (g/mol) | Mn  (g/mol) | PDI |
| --- | --- | --- | --- | --- | --- | --- | --- | --- | --- |
| P-100 | 27.78 | 32.98 | 8.99 | 7.47 | 2.88 | 19.90 | 1.58×10^4^ | 8.87×10^3^ | 1.78 |
| P-110 | 25.70 | 28.44 | 6.32 | 4.06 | 1.31 | 34.17 | 3.21×10^4^ | 1.18×10^4^ | 2.71 |
| P-120 | 25.31 | 24.96 | 6.30 | 3.10 | 1.02 | 39.32 | 5.80×10^4^ | 1.96×10^4^ | 2.96 |
| P-130 | 25.69 | 27.56 | 5.58 | 2.71 | 1.20 | 37.27 | 8.45×10^4^ | 3.25×10^4^ | 2.60 |
| P-140 | 26.55 | 33.05 | 2.24 | 2.82 | 0.08 | 35.26 | 4.67×10^4^ | 1.57×10^4^ | 2.97 |

**Table S2 The response values of different carbon structures in the samples**

| Samples | 284.6 eV | 286.6 eV | 289 eV |
| --- | --- | --- | --- |
|  | C-C/C-H | C-O-C/C-OH/C-S | O-C-O/=CO |
|  | response value  (a.u.) | response value  (a.u.) | response value  (a.u.) |
| P-100 | 678.18 | 1552.82 | 496.88 |
| P-110 | 688.71 | 2050.69 | 622.03 |
| P-120 | 524.57 | 3374.33 | 1041.28 |
| P-130 | 1188.79 | 2224.09 | 584.11 |
| P-140 | 1092.95 | 2416.18 | 686.32 |

**Table S3 PCR related primer sequences**

|  | Target Gene | Primer Sequences |
| --- | --- | --- |
|  | β-actin | Forward: 5’-TCTGGCATCACACCTTCTACAAT-3’  Reverse: 5’-TGTTGGCTTTGGGATTCAGG-3’ |
| Neurodevelopment-related genes | nauroD | Forward: 5’-CAGCAAGTGCTTCCTTTTCC-3’  Reverse: 5’-TAAGGGGTCCGTCAAATGAG-3’ |
|  | syn2a | Forward: 5’-GTGACCATGCCAGCATTTC -3’  Reverse: 5’-TGGTTCTCCACTTTCACCTT-3’ |
|  | mbp | Forward: 5’-AATCAGCAGGTTCTTCGGAGGAGA -3’  Reverse: 5’-AAGAAATGCACGACAGGGTTGACG-3’ |
|  | gap43 | Forward: 5’-TGCTGCATCAGAAGAACTAA-3’  Reverse: 5’-CCTCCGGTTTGATTCCATC-3’ |
|  | gfap | Forward: 5’-GGATGCAGCCAATCGTAAT-3’  Reverse: 5’-TTCCAGGTCACAGGTCAG-3’ |
|  | α1-tubulin | Forward: 5’-AATCACCAATGCTTGCTTCGAGCC-3’  Reverse: 5’-TTCACGTCTTTGGGTACCACGTCA-3’ |
| Oxidative stress-related genes | Mn-Sod | Forward: 5’- AATCAGCAGGTTCTTCGGAGGAGA-3’  Reverse: 5’- ACACTCGGTTGCTCTCTTTTCTCT-3’ |
|  | Cu/Zn-Sod | Forward: 5’- GTCGTCTGGCTTGTGGAGTG-3’  Reverse: 5’- TGTCAGCGGGCTAGTGCTT-3’ |
|  | cat | Forward: 5’- AGGGCAACTGGGATCTTACA-3’  Reverse: 5’- TTTATGGGACCAGACCTTGG-3’ |
| Neurotransmitter-related genes | *pet1* | Forward: 5’- CCATTCAGTTTTCAGGTATTTCC -3’  Reverse: 5’- GGCTGTGGTAGAGGGTTGGAG -3’ |
|  | *tph1b* | Forward: 5’- GTAAATCGAGACGGCGCAAC -3’  Reverse: 5’- GGCACCTCAGTAAGATCGCT -3’ |
|  | *htr3* | Forward: 5’- TTGTACGTCTGGTCCACAAGC -3’  Reverse: 5’- TGTTTTCCTTGTAATGCTCCAGGT -3’ |
|  | *htr4* | Forward: 5’- CCAACTATTTCATCGTGTCCCTG-3’  Reverse: 5’- GTTTCACCGTAGATCCAGTTCTC-3’ |

**Table S4 Data of 34 significantly differential genera of intestinal flora at the genus level in Zebrafish from different treatment groups**

| Genus | Control  _1 | Control  _2 | Control  _3 | Control  _4 | 0.1%  P-130  _1 | 0.1%  P-130  _2 | 0.1%  P-130  _3 | 0.1%  P-130  _4 | BPAF  _1 | BPAF  _2 | BPAF  _3 | BPAF  _4 | BPAF  +  0.05%  P-130  _1 | BPAF  +  0.05%  P-130  _2 | BPAF  +  0.05%  P-130  _3 | BPAF  +  0.05%  P-130  _4 | BPAF  +  0.1%  P-130  _1 | BPAF  +  0.1%  P-130  _2 | BPAF  +  0.1%  P-130  _3 | BPAF  +  0.1%  P-130  _4 | BPAF  +  0.5%  P-130  _1 | BPAF  +  0.5%  P-130  _2 | BPAF  +  0.5%  P-130  _3 | BPAF  +  0.5%  P-130  _4 |
| --- | --- | --- | --- | --- | --- | --- | --- | --- | --- | --- | --- | --- | --- | --- | --- | --- | --- | --- | --- | --- | --- | --- | --- | --- |
| Rodentibacter | 0.02 | 0.02 | 0.01 | 0.04 | 0 | 0 | 0 | 0 | 0 | 0 | 0 | 0 | 0 | 0 | 0 | 0 | 0 | 0 | 0 | 0 | 0 | 0 | 0 | 0 |
| Gemmataceae_  unclassified | 0.02 | 0.01 | 0.03 | 0.01 | 0 | 0 | 0.01 | 0 | 0 | 0 | 0 | 0 | 0 | 0 | 0 | 0 | 0.01 | 0.01 | 0.15 | 0.05 | 0 | 0 | 0 | 0 |
| CL500-29_  marine_group | 0.19 | 0.10 | 0.01 | 0.01 | 0 | 0 | 0.00 | 0 | 0 | 0 | 0 | 0 | 0 | 0 | 0 | 0 | 0.09 | 0.01 | 0.19 | 0.11 | 0 | 0 | 0 | 0.01 |
| Nocardia | 0.50 | 0.21 | 0.05 | 0.02 | 0 | 0 | 0.00 | 0 | 0 | 0 | 0.01 | 0 | 0 | 0 | 0 | 0 | 0.03 | 0.03 | 0.13 | 0.05 | 0.01 | 0.00 | 0.01 | 0 |
| Citrobacter | 0.67 | 0.22 | 0.10 | 0.37 | 0 | 0 | 0 | 0 | 0.09 | 0.15 | 1.43 | 0.12 | 0.03 | 0 | 0.01 | 0 | 0.09 | 0.06 | 0.08 | 0.25 | 0.06 | 0.12 | 0.11 | 0.09 |
| ZOR0006 | 1.20 | 1.03 | 0.53 | 0.69 | 0 | 0 | 0.01 | 0.01 | 0.29 | 0.38 | 0.85 | 1.10 | 0.19 | 0.24 | 0.12 | 0.07 | 0.18 | 0.19 | 0.44 | 0.16 | 0.47 | 0.16 | 0.12 | 0.20 |
| Comamonas | 0.01 | 0 | 0.01 | 0.03 | 0 | 0 | 0 | 0 | 0 | 0 | 0 | 0 | 0 | 0 | 0 | 0 | 0 | 0 | 0 | 0 | 0 | 0 | 0 | 0 |
| Solobacterium | 0.01 | 0.01 | 0 | 0.01 | 0 | 0 | 0 | 0 | 0 | 0 | 0 | 0 | 0 | 0 | 0 | 0 | 0 | 0 | 0 | 0 | 0 | 0 | 0 | 0 |
| Bacteroidota_  unclassified | 0.39 | 3.89 | 3.58 | 2.38 | 0 | 0.07 | 0.17 | 0.05 | 7.53 | 8.21 | 21.12 | 7.86 | 3.26 | 2.50 | 7.12 | 0.71 | 3.17 | 5.27 | 1.50 | 1.33 | 0.80 | 1.42 | 1.43 | 5.23 |
| Vibrio | 0.01 | 0.03 | 0.14 | 0.08 | 93.44 | 94.84 | 1.21 | 94.00 | 0.07 | 0.42 | 0.24 | 0.31 | 4.32 | 0.20 | 0.69 | 1.07 | 0.26 | 0.01 | 0.06 | 0 | 0.05 | 9.72 | 9.53 | 0.59 |
| Cetobacterium | 73.42 | 61.30 | 60.60 | 68.59 | 0.18 | 0.73 | 37.48 | 0.59 | 50.96 | 45.62 | 40.72 | 52.44 | 55.15 | 68.88 | 57.47 | 40.18 | 61.78 | 63.27 | 76.10 | 75.35 | 74.78 | 17.26 | 17.74 | 39.73 |
| Akkermansia | 1.75 | 1.97 | 1.01 | 2.49 | 0.07 | 0.23 | 0.11 | 0.13 | 4.16 | 1.16 | 4.94 | 2.70 | 0.53 | 0.29 | 1.16 | 0.80 | 0.69 | 0.69 | 0.96 | 0.38 | 2.07 | 0.08 | 0.34 | 1.52 |
| Odoribacter | 0.01 | 0.01 | 0.01 | 0.01 | 0.01 | 0.01 | 0.02 | 0.01 | 0.01 | 0 | 0 | 0.01 | 0.00 | 0 | 0 | 0 | 0 | 0 | 0 | 0 | 0 | 0 | 0 | 0.00 |
| Erysipelotrichaceae  _unclassified | 0.03 | 0.01 | 0.01 | 0.03 | 0.01 | 0 | 0 | 0 | 0.01 | 0 | 0 | 0.01 | 0 | 0 | 0 | 0 | 0 | 0 | 0.01 | 0 | 0 | 0 | 0 | 0 |
| Rikenellaceae  _unclassified | 0.06 | 0.87 | 0.79 | 0.42 | 0.01 | 0 | 0 | 0 | 1.36 | 1.79 | 4.61 | 1.59 | 0.66 | 0.42 | 1.46 | 0 | 0.63 | 1.23 | 0.27 | 0.29 | 0.10 | 0.49 | 0.42 | 1.22 |
| Bacteroides | 0.16 | 0.66 | 0.50 | 0.21 | 0.03 | 0.03 | 0.02 | 0.02 | 0.85 | 1.24 | 0.94 | 1.49 | 0.27 | 0.11 | 0.23 | 0.01 | 0.48 | 0.31 | 0.09 | 0.06 | 0.01 | 0.22 | 0.21 | 0.42 |
| Barnesiellaceae  _unclassified | 0.35 | 5.18 | 3.53 | 10.52 | 0.01 | 0.06 | 0.05 | 0.04 | 15.12 | 19.00 | 6.89 | 10.76 | 11.28 | 5.79 | 8.34 | 0.22 | 8.85 | 6.10 | 2.75 | 2.79 | 0.38 | 1.72 | 1.64 | 8.60 |
| Streptomyces | 0.00 | 0 | 0.01 | 0.01 | 0 | 0 | 0 | 0 | 0 | 0 | 0.00 | 0 | 0 | 0 | 0 | 0 | 0 | 0 | 0 | 0 | 0.01 | 0 | 0.01 | 0.03 |
| Prevotellaceae_UCG-001 | 0.00 | 0.01 | 0.02 | 0.00 | 0.01 | 0 | 0 | 0 | 0 | 0 | 0 | 0 | 0 | 0.00 | 0 | 0 | 0 | 0 | 0 | 0 | 0 | 0 | 0 | 0.00 |
| Desulfovibrionaceae  _unclassified | 0.01 | 0.01 | 0.01 | 0.02 | 0.10 | 0.08 | 0.04 | 0.09 | 0.11 | 0.10 | 0.07 | 0.07 | 0.05 | 0.05 | 0.10 | 0.05 | 0.08 | 0.10 | 0.11 | 0.08 | 0.07 | 0 | 0.05 | 0.08 |
| Rothia | 0 | 0 | 0 | 0 | 0.00 | 0 | 0.00 | 0.01 | 0.01 | 0 | 0 | 0 | 0 | 0.01 | 0.03 | 0.03 | 0.03 | 0.02 | 0.03 | 0.03 | 0.03 | 0 | 0.03 | 0 |
| Sarcina | 0 | 0 | 0 | 0 | 0 | 0.03 | 0.04 | 0.05 | 0 | 0.06 | 0.05 | 0.04 | 0.03 | 0.03 | 0 | 0 | 0 | 0 | 0 | 0 | 0 | 0 | 0 | 0 |
| Lachnospiraceae  _XPB1014_group | 0 | 0 | 0 | 0 | 0 | 0 | 0.01 | 0 | 0 | 0.02 | 0.00 | 0 | 0.01 | 0.01 | 0.01 | 0.00 | 0.00 | 0 | 0 | 0 | 0 | 0 | 0 | 0 |
| UCG-010_unclassified | 0 | 0 | 0 | 0 | 0.01 | 0.00 | 0 | 0 | 0.00 | 0 | 0.00 | 0.00 | 0 | 0 | 0 | 0 | 0 | 0 | 0 | 0 | 0 | 0 | 0 | 0 |
| Muribaculum | 0.04 | 0.02 | 0.03 | 0.05 | 0.05 | 0.03 | 0.02 | 0.02 | 0.02 | 0 | 0.03 | 0.01 | 0.02 | 0.02 | 0.01 | 0.00 | 0 | 0 | 0.06 | 0.00 | 0 | 0 | 0.00 | 0 |
| Gemmata | 0.06 | 0.08 | 0.01 | 0.03 | 0 | 0 | 0.03 | 0 | 0 | 0.02 | 0 | 0 | 0.01 | 0.01 | 0 | 0.04 | 0.12 | 0.02 | 0.17 | 0.07 | 0 | 0.01 | 0 | 0.02 |
| Verrucomicrobiae  _unclassified | 0 | 0.06 | 0.03 | 0 | 0 | 0 | 0 | 0 | 0.02 | 0.02 | 0 | 0.01 | 0 | 0 | 0 | 0 | 0 | 0 | 0 | 0 | 0 | 0 | 0 | 0 |
| Anaerotruncus | 0.07 | 0.05 | 0.04 | 0.02 | 0.02 | 0.01 | 0 | 0.01 | 0.02 | 0.01 | 0.01 | 0.01 | 0.01 | 0.02 | 0 | 0.00 | 0 | 0.01 | 0 | 0.01 | 0 | 0 | 0 | 0.02 |
| Eubacterium]_  coprostanoligenes_  group_unclassified | 0 | 0 | 0.01 | 0 | 0.05 | 0.01 | 0.01 | 0.01 | 0.07 | 0.01 | 0.01 | 0.02 | 0.02 | 0.00 | 0.03 | 0.02 | 0.03 | 0.04 | 0.11 | 0.03 | 0.03 | 0 | 0.02 | 0 |
| Paludibacter | 0 | 0.22 | 0.16 | 0.09 | 0 | 0 | 0 | 0 | 0.21 | 0.26 | 1.17 | 0.31 | 0.23 | 0.17 | 0.21 | 0 | 0.12 | 0.36 | 0.05 | 0.06 | 0 | 0.13 | 0.13 | 0.29 |
| Turicibacter | 0 | 0 | 0.01 | 0 | 0.01 | 0.05 | 0.05 | 0.04 | 0.00 | 0.04 | 0.05 | 0.03 | 0.06 | 0.04 | 0.02 | 0.01 | 0.01 | 0.02 | 0.13 | 0.03 | 0.01 | 0 | 0.01 | 0.02 |
| Dysgonomonadaceae  _unclassified | 0 | 0 | 0 | 0 | 0 | 0 | 0 | 0 | 0.08 | 0.16 | 0.23 | 0 | 0 | 0 | 0.01 | 0 | 0 | 0.08 | 0 | 0 | 0 | 0.08 | 0.05 | 0.09 |
| cvE6_unclassified | 0 | 0 | 0 | 0 | 0 | 0 | 0.00 | 0 | 0 | 0 | 0 | 0 | 0 | 0 | 0 | 0 | 0 | 0 | 0.00 | 0 | 0.02 | 0.02 | 0.02 | 0 |
| Aquitalea | 0 | 0 | 0.01 | 0 | 0 | 0.00 | 2.62 | 0.02 | 0.01 | 0.01 | 0.01 | 0 | 0.16 | 0.39 | 0.06 | 0.31 | 0.07 | 0.04 | 0.10 | 0.10 | 0 | 0.42 | 0.43 | 0.04 |

**Ⅱ. Supplementary Figures Caption**

**Figure S1** FTIR spectra (a) and XPS response value (b) of different pectin from pomelo peels.

**Figure S2** XPS spectrum of different pectin samples: P-100 (a), P-110 (b), P-120 (c), P-130 (d), P-140 (e).

**Figure S3** DPPH scavenging rate (a), ·OH scavenging rate (b), and H₂O₂ scavenging rate (c) of pectin.

**Figure S4** The gene expression changes in zebrafish caused by different pectins after BPAF exposure. (a) *neuro-D*, (b) *α1-tubulin*, (c) *gfap*, (d) *syn2a*, (e) shha, (f) gap43, (g) *Mn-sod*, (h) *Cu/Zn-sod*, (i) *cat*.

**Figure S5** Heat map of neurotransmitter contents in different treatment groups.

**Figure S6** Analysis of alpha diversity of intestinal microbiota in different treatment groups. (a) Observed otus, (b) Chao1, (c) Simpson, (d) Shannon, (e) Pielou e.

**Figure S7** Abundance of *Cetobacterium* in different treatment groups.


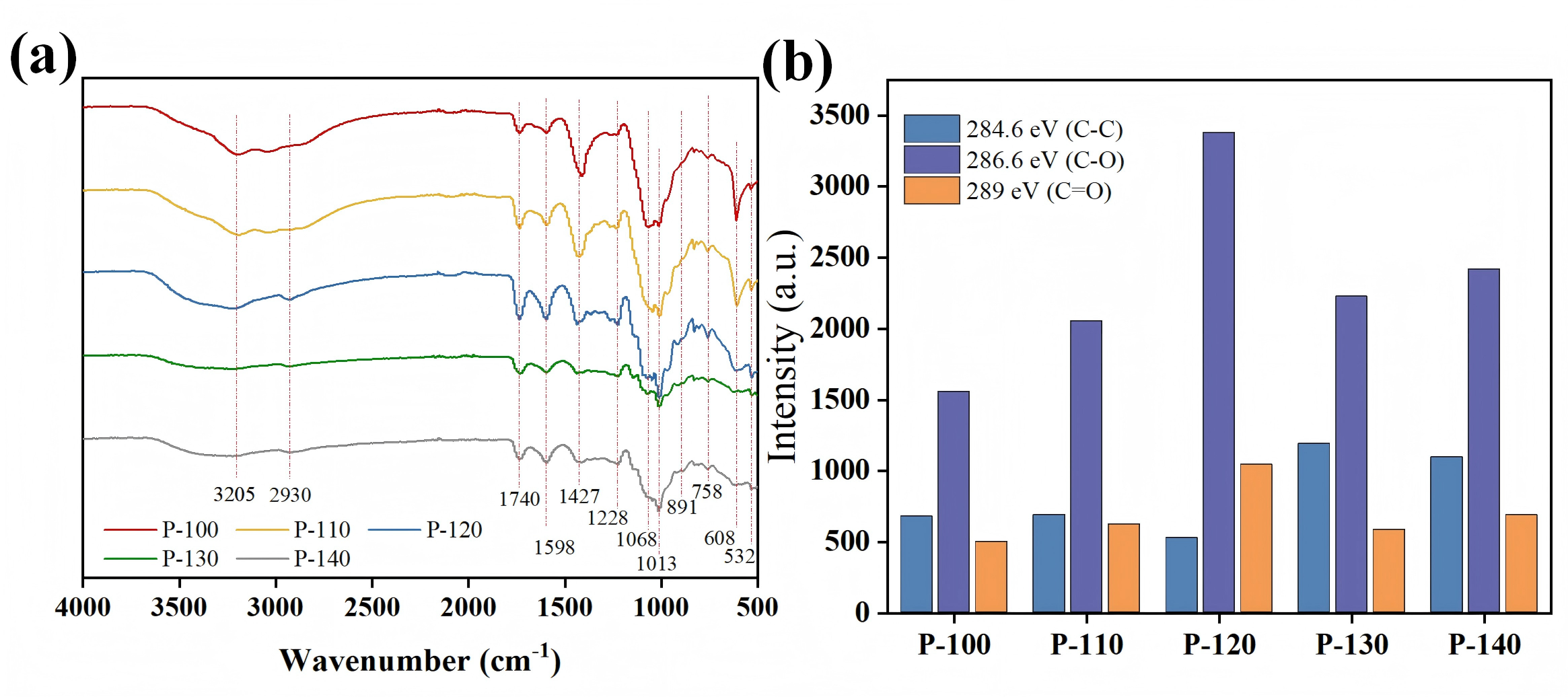


**Figure S1**

**
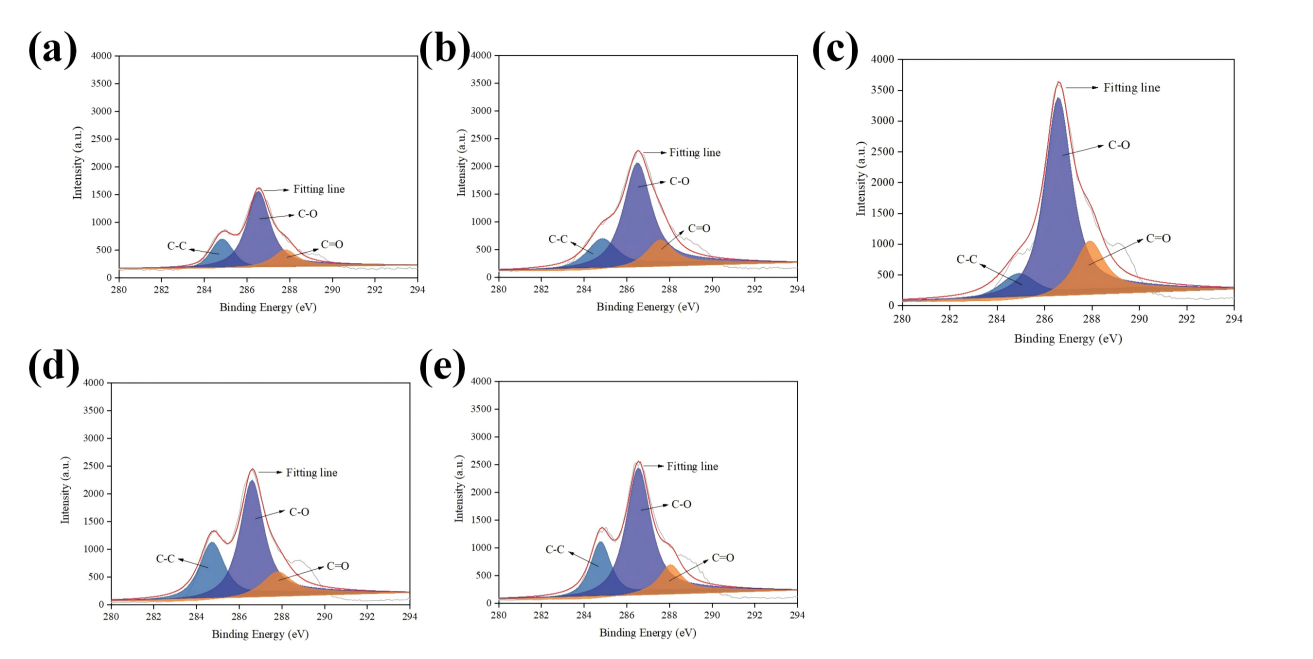
**

**Figure S2**

**
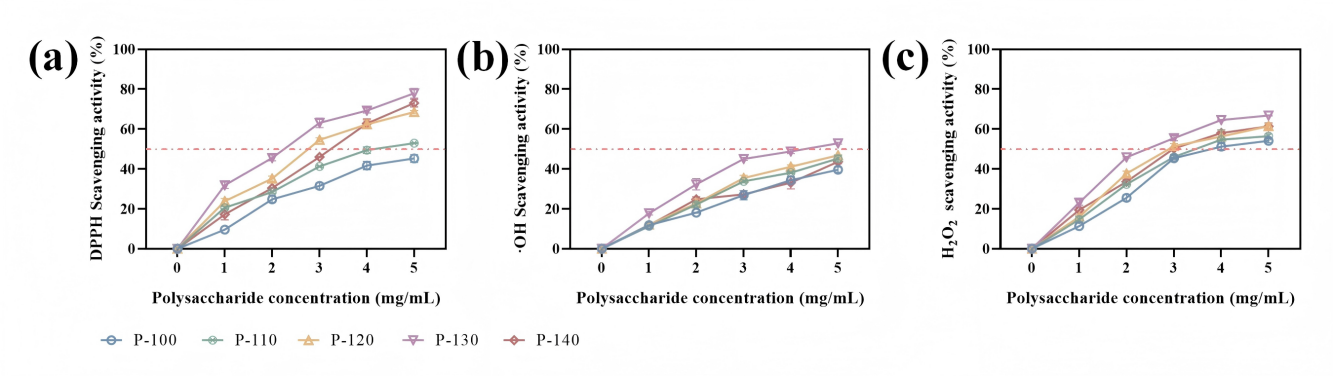
**

**Figure S3**

**
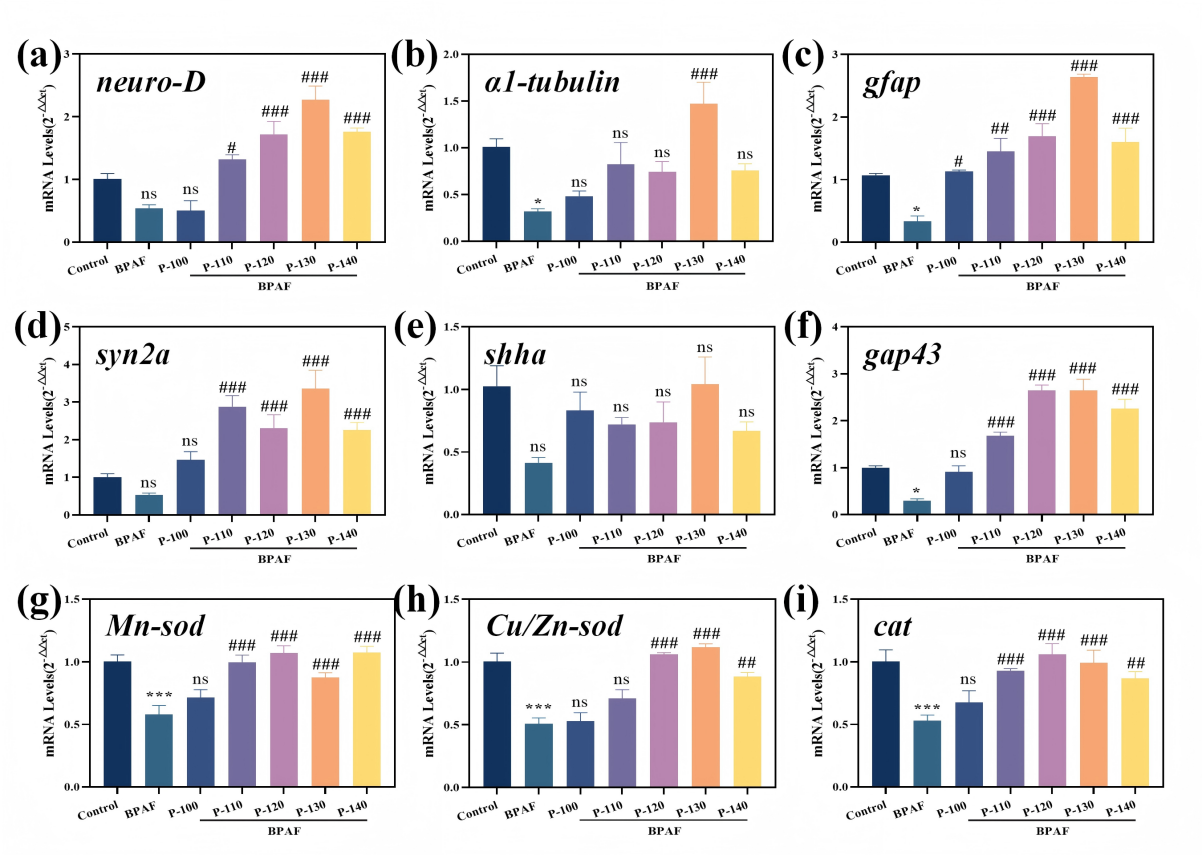
**

**Figure S4**

**
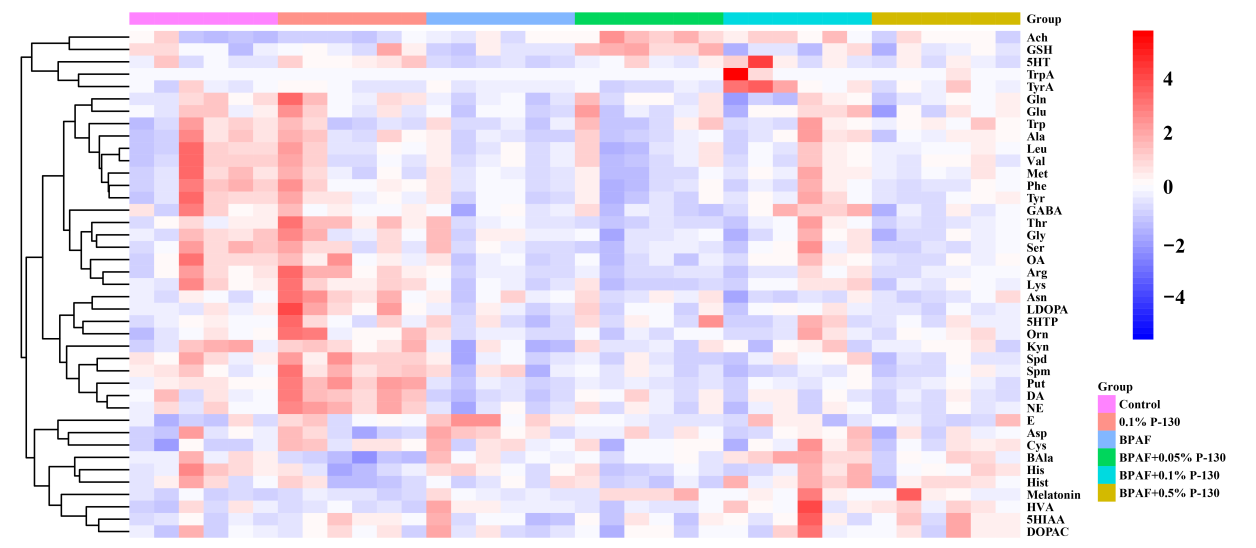
**

**Figure S5**

**
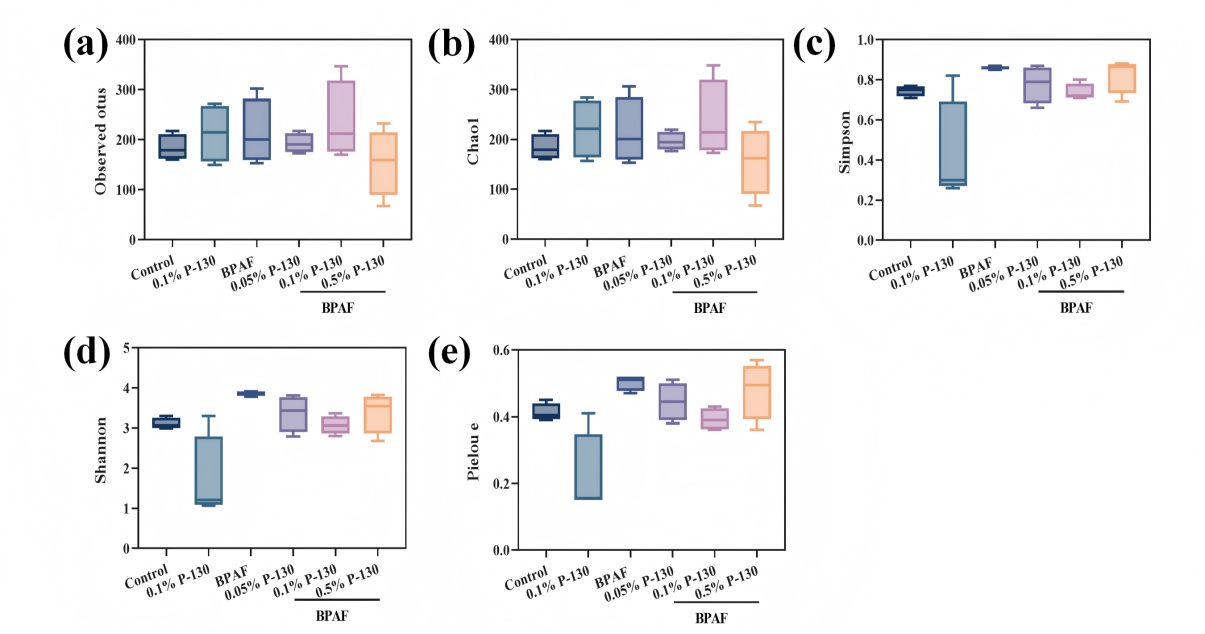
**

**Figure S6**

**
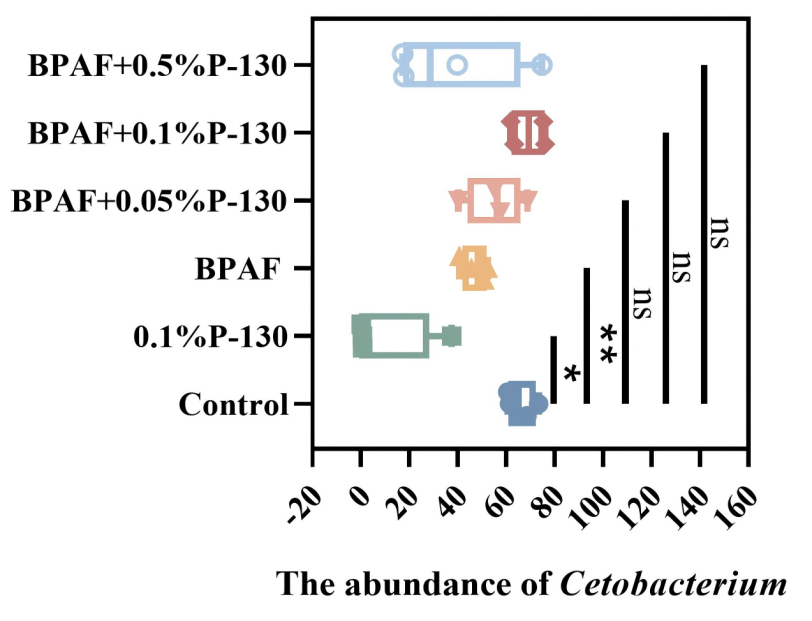
**

**Figure S7**
